# Supplementary material for: Identification and differential expression of piRNAs in the gonads of Amur sturgeon (Acipenser schrenckii)
Source: PeerJ. 2019 Apr 12;7:e6709. doi: 10.7717/peerj.6709 (PMC6499119; doi:10.7717/peerj.6709)
Supplement: Supplemental Information 1 — Figure S1. The sequence length distribution and frequency percentages of small RNA reads of Acipenser schrenckii. The x-axis indicates the lengths of small RNA reads. The y-axis indicates the percentages of small RNA reads with a specific length. Different colors suggest different types of small RNAs. (A) and (B), Ovary 1 and 2; (C) and (D), Testis 1 and 2. Figure S2. Expression pattern of sex-biased putative piRNAs validated by microarray. The expression of 311 sex-biased putative piRNAs is reflected as Log2FC. The heat map showed the putative piRNAs clustered into ten clades based on sexes, including five clades up-regulated in ovaries and five up-regulated in testes. For detailed information see Table S5. Table S1. Summary of library quality control and the ambiguous reads filter. Table S2. List of orthologs to known piRNAs. Table S3. Summary of 264 enriched GO annotations and 29 enriched KEGG pathways that may associate with sturgeon putative piRNA. *: Enriched GO/KEGG terms may involve in reproduction and aquaculture. Table S4. Summary of 130 enriched GO annotations and 2 enriched KEGG pathways that may associate with sex-specific putative piRNA. *: Enriched GO terms may involve in reproduction and aquaculture. Table S5. Validation of the expression of 311 sex-biased putative piRNAs by miccroarray. [file peerj-07-6709-s001.pdf]

Discovery and Characterization of piRNAs in male and female gonads of Amur sturgeon (*Acipenser schrenckii*)

Lihong Yuan, Linmiao Li, Xiujuan Zhang, Jinping Chen*

Figure S1 - The sequence length distribution and frequence percentage of small RNA reads of *Acipenser schrenckii*. The x-axis indicates the length of small RNA reads. The y-axis indicates the percentage of small RNA reads with specific length. Different color suggests different type of small RNAs. A) and B), Ovary 1 and 2; C) and D), Testis 1 and 2.

A

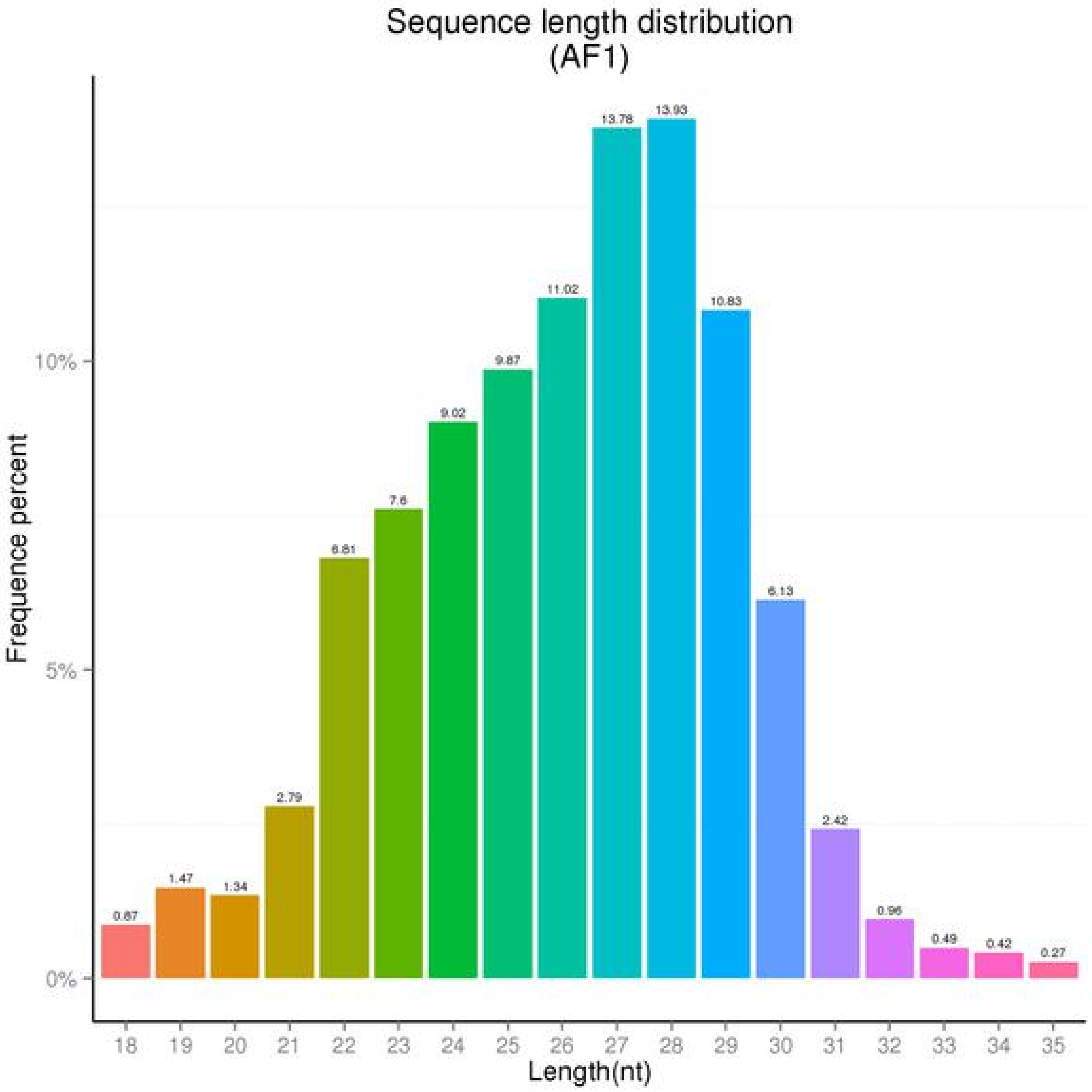

B

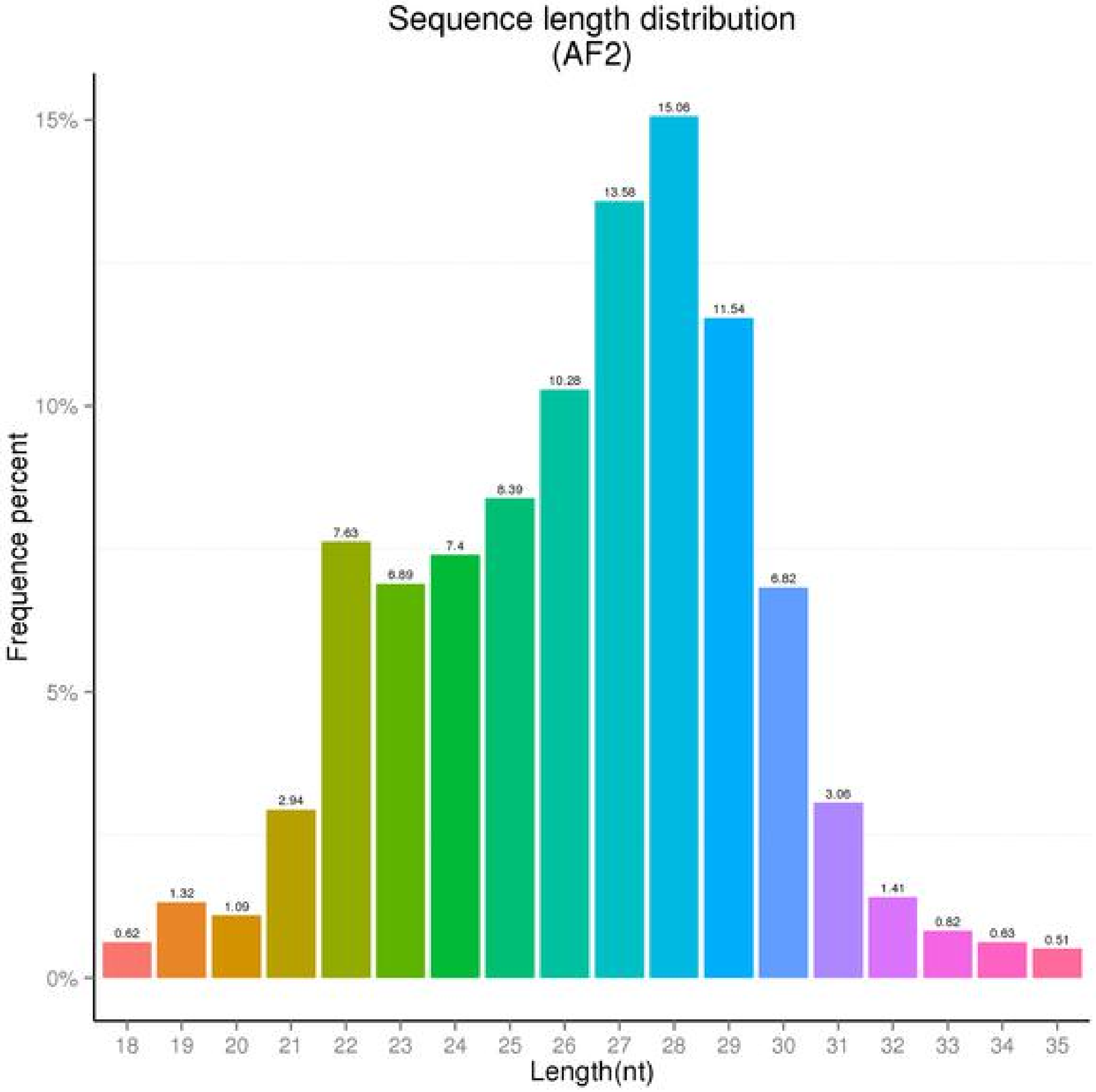

Sequence length distribution
(AF2)
Frequence percent
15%
10%
5%
0%
0.62
1.32
1.09
2.94
7.63
6.89
7.4
8.39
10.28
13.58
15.06
11.54
6.82
3.06
1.41
0.82
0.63
0.51
18
19
20
21
22
23
24
25
26
27
28
29
30
31
32
33
34
35
Length(nt)

C

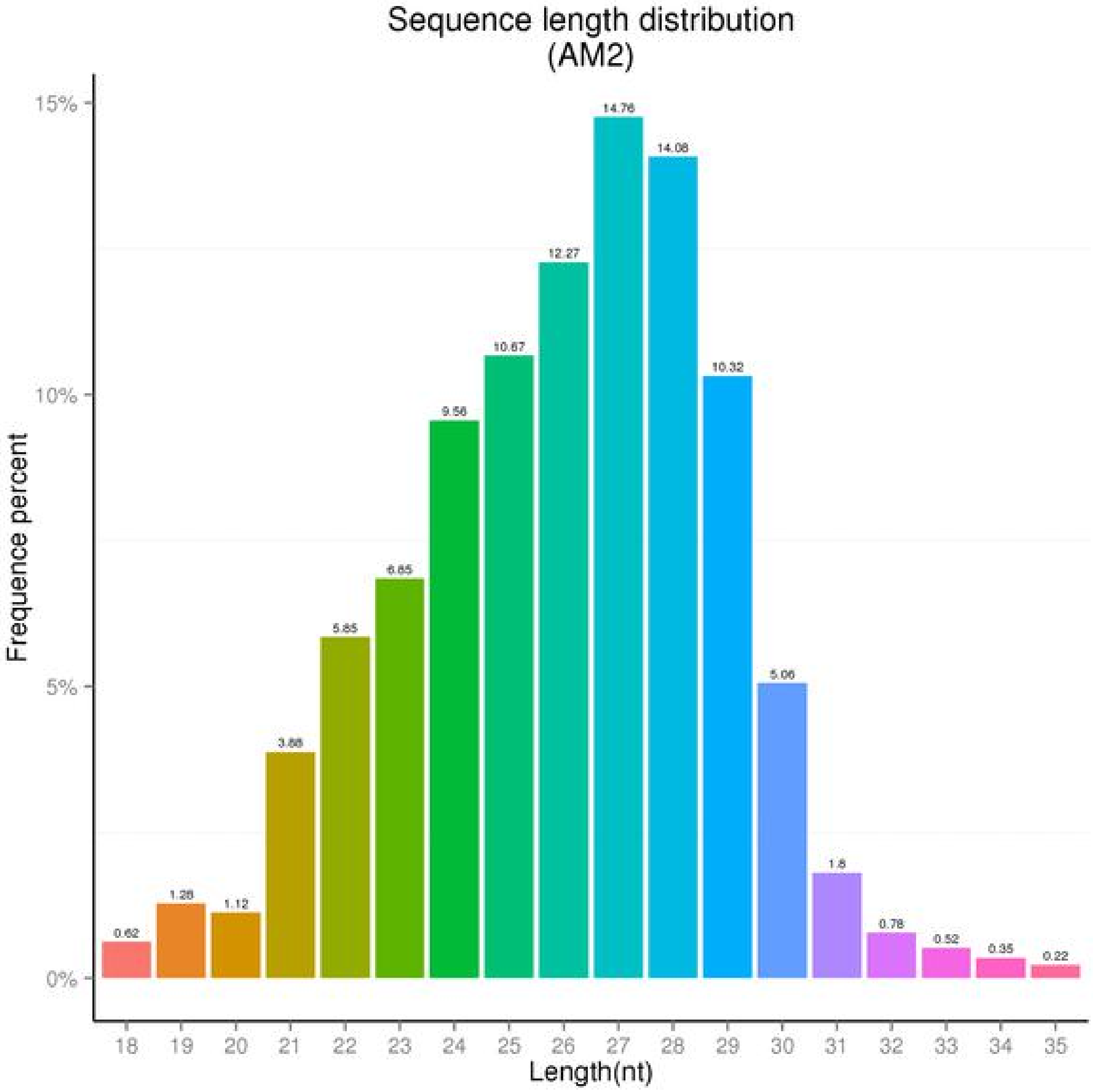

Sequence length distribution
(AM2)
15%
10%
5%
0%
Frequence percent
0.62
1.28
1.12
3.88
5.85
6.85
9.56
10.67
12.27
14.76
14.08
10.32
5.06
1.8
0.78
0.52
0.35
0.22
18
19
20
21
22
23
24
25
26
27
28
29
30
31
32
33
34
35
Length(nt)

D

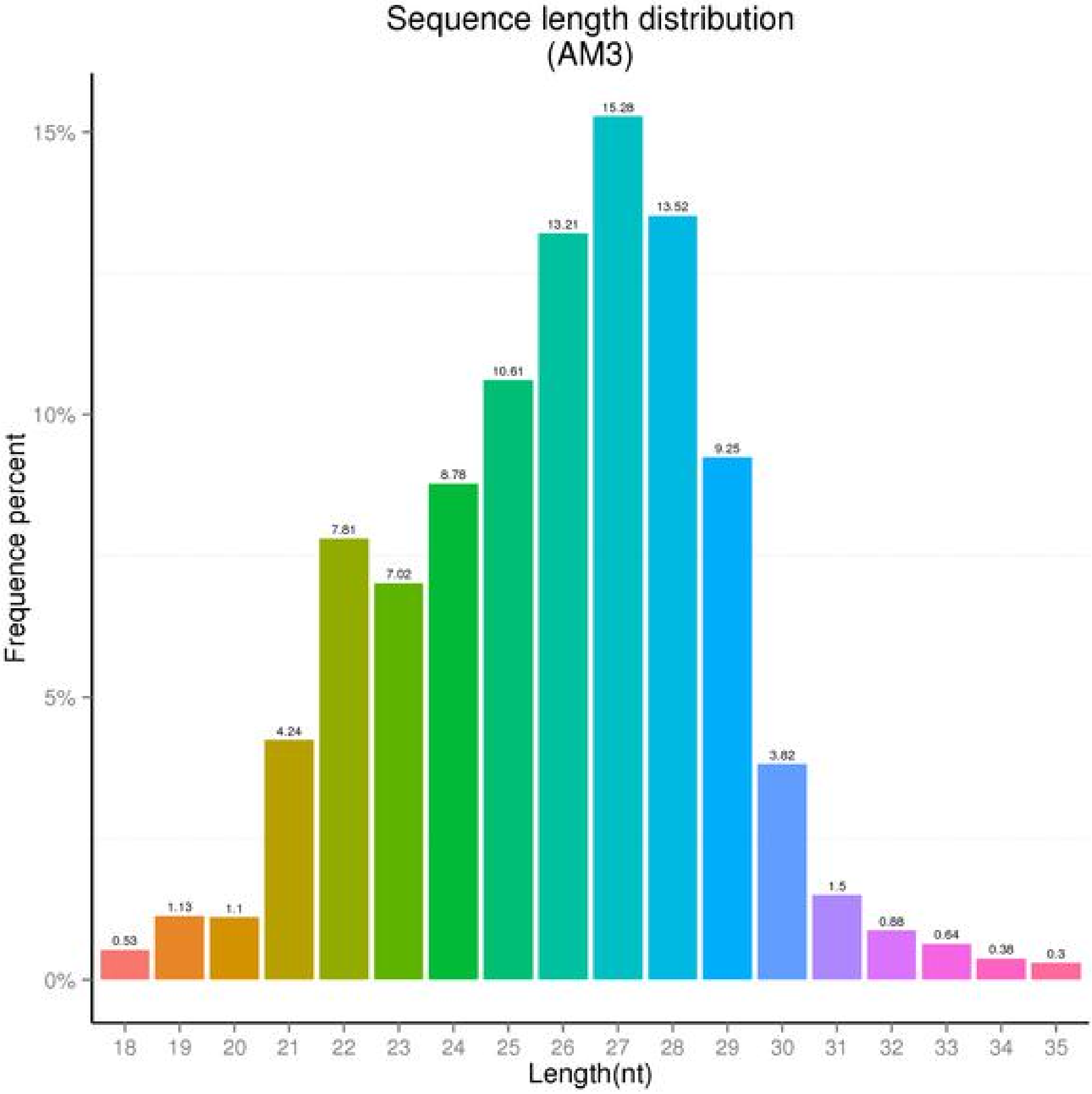

Sequence length distribution
(AM3)
Frequence percent
15%
10%
5%
0%
0.53
1.13
1.1
4.24
7.81
7.02
8.78
10.61
13.21
15.28
13.52
9.25
3.82
1.5
0.88
0.64
0.38
0.3
18
19
20
21
22
23
24
25
26
27
28
29
30
31
32
33
34
35
Length(nt)

Figure S2. Expression pattern of sex-biased putative piRNAs validated by microarray. The expression of 311 sex-biased putative piRNAs is reflected as Log2FC. The heat map showed the putative piRNAs clustered into ten clades based on sexes, including five clades up-regulated in ovaries and five up-regulated in testes. For detailed information see Table S5.

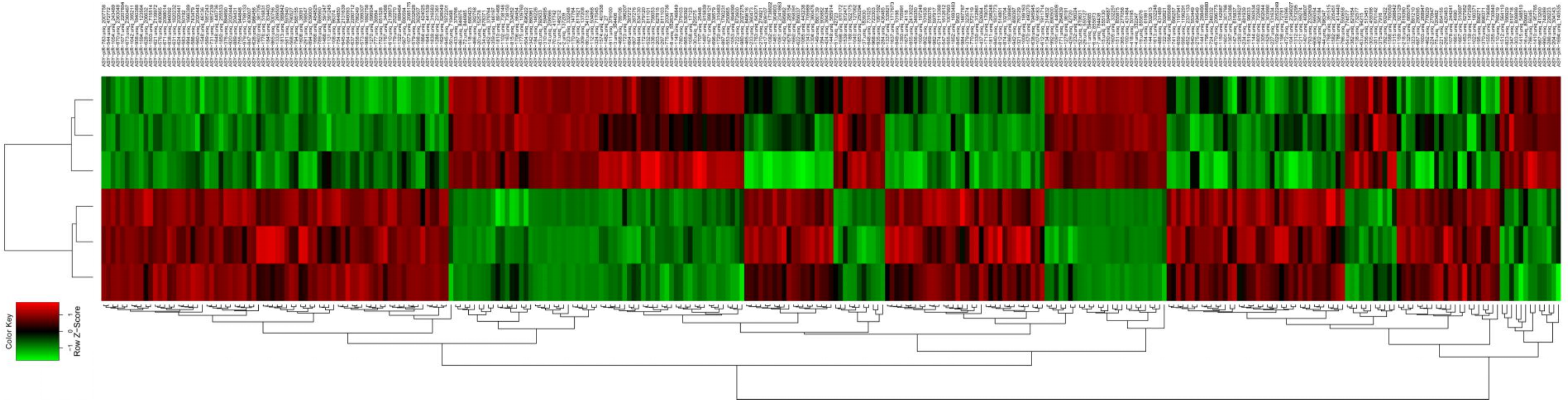

Table S1 - Summary of library quality control and the ambiguous reads filter.

Sample ID	Sample description	Reads	Bases	Error rate	Q20	Q30	GC content	N% > 10%	low quality	5 adapter contamine	3 adapter null or insert null	with ployA/T/G/C	clean reads	Total sRNA	Mapped sRNA	"+" Mapped sRNA	"-" Mapped sRNA
**AF1**	**Ovary 1**	8374222 (100.00%)	0.419G	0.01%	98.61%	97.28%	47.74%	20 (0.00%)	775 (0.01%)	1251 (0.01%)	347039 (4.14%)	47700 (0.57%)	7977437 (95.26%)	7719149 (92.18%)	3367185 (40.21%)	1663800 (19.87%)	1703385 (20.34%)
**AF2**	**Ovary 2**	7762049 (100.00%)	0.388G	0.01%	98.63%	97.29%	48.10%	26 (0.00%)	703 (0.01%)	1224 (0.02%)	388225 (5.00%)	40969 (0.53%)	7330902 (94.45%)	7012600 (90.34%)	3303306 (42.56%)	1661237 (21.40%)	1642069 (21.16%)
**AM2**	**Testis 1**	7717550 (100.00%)	0.386G	0.01%	98.71%	97.44%	48.13%	23 (0.00%)	657 (0.01%)	1005 (0.01%)	317293 (4.11%)	42664 (0.55%)	7355908 (95.31%)	7129325 (92.39%)	3164300 (41.00%)	1583153 (20.51%)	1581147 (20.49%)
**AM3**	**Testis 2**	8557963 (100.00%)	0.428G	0.01%	98.65%	97.32%	48.03%	25 (0.00%)	723 (0.01%)	1245 (0.01%)	451253 (5.27%)	48639 (0.57%)	8056078 (94.14%)	7803509 (91.18%)	3126400 (36.53%)	1550844 (18.12%)	1575556 (18.41%)

Table S2 - List of orthologs to known piRNAs

piRNA	piRNA sequence	known piRNA	known piRNA sequence	known piRNA description
uniq_231483	TCTGCCCAGTGCTCTGAATGTCAAAGT	dme_piR_009519	TCTGCCCAGTGCTCTGAATGTCAAAGTG	Drosophila_melanogaster:U:825171:825198:Plus
uniq_1332959	TCTGCCCAGTGCTCTGAATGTCAAA	dme_piR_009519	TCTGCCCAGTGCTCTGAATGTCAAAGTG	Drosophila_melanogaster:U:825171:825198:Plus
uniq_1596260	CTGCCCAGTGCTCTGAATGTCAAAGT	dme_piR_009519	TCTGCCCAGTGCTCTGAATGTCAAAGTG	Drosophila_melanogaster:U:825171:825198:Plus
uniq_2785878	GCATCGGTGGTTCAGTGGTAGAATGC	dme_piR_010919	GCATCGGTGGTTCAGTGGTAGAATGCTC	Drosophila_melanogaster:2L:2031692:2031719:Plus
uniq_519316	TGGGAATACCAGGTGCTGTAAGCTA	dre_piR_0001123	TGGGAATACCAGGTGCTGTAAGCTA	Danio_rerio:4:38948896:38948920:Minus
uniq_783379	TAGACAGCAGGACGGTGGCCATGGA	dre_piR_0002388	TAGACAGCAGGACGGTGGCCATGGAAGTC	Danio_rerio:14:34501105:34501129:Minus
uniq_1641194	AGACAGCAGGACGGTGGCCATGGAAGT	dre_piR_0002388	TAGACAGCAGGACGGTGGCCATGGAAGTC	Danio_rerio:14:34501105:34501129:Minus
uniq_2370819	AGACAGCAGGACGGTGGCCATGGAAGTC	dre_piR_0002388	TAGACAGCAGGACGGTGGCCATGGAAGTC	Danio_rerio:14:34501105:34501129:Minus
uniq_521643	GCATTGGTGGTTCAGTGGTAGAATTCTCGC	dre_piR_0002593	GCATTGGTGGTTCAGTGGTAGAATTCTCGC	Danio_rerio:6:47439166:47439190:Minus
uniq_738146	GCATTGGTGGTTCAGTGGTAGAATTCTC	dre_piR_0002594	GCATTGGTGGTTCAGTGGTAGAATTCTCG	Danio_rerio:24:891219:891243:Plus
uniq_296661	GCATTGGTGGTTCAGTGGTAGAATTCT	dre_piR_0002595	GCATTGGTGGTTCAGTGGTAGAATTCTC	Danio_rerio:8:39868444:39868472:Plus
uniq_82822	TGGGAATACCAGGTGCTGTGAGCTT	dre_piR_0004353	TGGGAATACCAGGTGCTGTGAGCTT	Danio_rerio:4:37882405:37882429:Minus
uniq_2400385	ACCCGAAAGATGGTGAACTATGCCTGGGC	dre_piR_0006070	ACCCGAAAGATGGTGAACTATGCCTGGGC	Danio_rerio:6:31619152:31619176:Plus
uniq_3024074	CCGAAAGATGGTGAACTATGCCTGGGC	dre_piR_0006070	ACCCGAAAGATGGTGAACTATGCCTGGGC	Danio_rerio:6:31619152:31619176:Plus
uniq_2097900	GAAAGATGGTGAACTATGCCTGGGC	dre_piR_0006070	ACCCGAAAGATGGTGAACTATGCCTGGGC	Danio_rerio:6:31619152:31619176:Plus
uniq_295323	CCGAAAGATGGTGAACTATGCCTGGG	dre_piR_0012448	ACCCGAAAGATGGTGAACTATGCCTGGG	Danio_rerio:12:12717727:12717750:Plus
uniq_941207	TTGGTGAATTCTGCTTCACAATGACA	dre_piR_0013017	TTGGTGAATTCTGCTTCACAATGACAGG	Danio_rerio:21:16628981:16629004:Minus
uniq_606128	TTGGTGAATTCTGCTTCACAATGAC	dre_piR_0013019	TTGGTGAATTCTGCTTCACAATGACAG	Danio_rerio:10:8985377:8985400:Minus
uniq_1114509	CTACCATCTGTGGGATTATGACTGAACGC	dre_piR_0016672	CTACCATCTGTGGGATTATGACTGAACGC	Danio_rerio:5:64168419:64168447:Plus
uniq_442139	TACCATCTGTGGGATTATGACTGAACGC	dre_piR_0016672	CTACCATCTGTGGGATTATGACTGAACGC	Danio_rerio:5:64168419:64168447:Plus
uniq_295268	TGGTGAACTATGCCTGGGCAGGGCGAA	dre_piR_0019149	TGGTGAACTATGCCTGGGCAGGGCGAAG	Danio_rerio:5:64165746:64165773:Plus
uniq_457625	GCATTGGTGGTTCAGTGGTAGAATTCTCGCC	dre_piR_0029993	GCATTGGTGGTTCAGTGGTAGAATTCTCGCCT	Danio_rerio:21:34775365:34775396:Minus
uniq_1968022	TGGTGGTTCAGTGGTAGAATTCTCGCCT	dre_piR_0029993	GCATTGGTGGTTCAGTGGTAGAATTCTCGCCT	Danio_rerio:21:34775365:34775396:Minus
uniq_962627	TTGGTGGTTCAGTGGTAGAATTCTCGCCT	dre_piR_0029993	GCATTGGTGGTTCAGTGGTAGAATTCTCGCCT	Danio_rerio:21:34775365:34775396:Minus
uniq_800856	TGGTGGTTCAGTGGTAGAATTCTCGCC	dre_piR_0029993	GCATTGGTGGTTCAGTGGTAGAATTCTCGCCT	Danio_rerio:21:34775365:34775396:Minus
uniq_258265	TTGGTGGTTCAGTGGTAGAATTCTCGCC	dre_piR_0029993	GCATTGGTGGTTCAGTGGTAGAATTCTCGCCT	Danio_rerio:21:34775365:34775396:Minus
uniq_2611026	TCCGAAGTTTCCCTCAGGATAGCTGG	dre_piR_0031044	TCCGAAGTTTCCCTCAGGATAGCTGGCG	Danio_rerio:5:64165882:64165909:Plus
uniq_77832	CCCGAAAGATGGTGAACTATGCCTGG	dre_piR_0032495	ACCCGAAAGATGGTGAACTATGCCTGG	Danio_rerio:5:64165736:64165762:Plus
uniq_142422	AGCAGGACGGTGGCCATGGAAGTCGGC	dre_piR_0035551	AGCAGGACGGTGGCCATGGAAGTCGGC	Danio_rerio:4:39613435:39613461:Minus
uniq_707926	TTGGTGGTTCAGTGGTAGAATTCTC	dre_piR_0037750	GCATTGGTGGTTCAGTGGTAGAATTCTCGCC	Danio_rerio:21:34775366:34775396:Minus
uniq_334052	GCATTGGTGGTTCAGTGGTAGAATTCTCG	dre_piR_0037750	GCATTGGTGGTTCAGTGGTAGAATTCTCGCC	Danio_rerio:21:34775366:34775396:Minus
uniq_524388	GCATTGGTGGTTCAGTGGTAGAATTC	dre_piR_0037750	GCATTGGTGGTTCAGTGGTAGAATTCTCGCC	Danio_rerio:21:34775366:34775396:Minus
uniq_845610	TTGGTGGTTCAGTGGTAGAATTCTCG	dre_piR_0037750	GCATTGGTGGTTCAGTGGTAGAATTCTCGCC	Danio_rerio:21:34775366:34775396:Minus
uniq_2505226	GCATTGGTGGTTCAGGGGTAGAATTCT	dre_piR_0043343	GCATTGGTGGTTCAGGGGTAGAATTCTCGC	Danio_rerio:14:34777330:34777359:Minus
uniq_2073500	CCCTGTTGAGCTTGACTCTAGTCTGGC	dre_piR_0045878	ACCCTGTTGAGCTTGACTCTAGTCTGGCCC	Danio_rerio:4:34937644:34937673:Minus
uniq_2208829	CCTGTTGAGCTTGACTCTAGTCTGGC	dre_piR_0045878	ACCCTGTTGAGCTTGACTCTAGTCTGGCCC	Danio_rerio:4:34937644:34937673:Minus
uniq_2423014	GCATTGGTGGTTCAGTGGTAGAATGC	dre_piR_0047433	AGCATTGGTGGTTCAGTGGTAGAATGC	Danio_rerio:5:42102943:42102969:Plus

uniq_12	TGATGATGATGATGATGATGCTGATG	dre_piR_0053487	TGATGATGATGATGATGATGCTGATG	Danio_rerio:18:3129142:3129167:Plus
uniq_2325806	TTATGACTGAACGCCTCTAAGTCAGAA	dre_piR_0055038	GATTATGACTGAACGCCTCTAAGTCAGAAT	Danio_rerio:5:64168432:64168461:Plus
uniq_689009	TTATGACTGAACGCCTCTAAGTCAGA	dre_piR_0055038	GATTATGACTGAACGCCTCTAAGTCAGAAT	Danio_rerio:5:64168432:64168461:Plus
uniq_1887516	ATGACTGAACGCCTCTAAGTCAGAAT	dre_piR_0055038	GATTATGACTGAACGCCTCTAAGTCAGAAT	Danio_rerio:5:64168432:64168461:Plus
uniq_2412492	TATGACTGAACGCCTCTAAGTCAGAAT	dre_piR_0055038	GATTATGACTGAACGCCTCTAAGTCAGAAT	Danio_rerio:5:64168432:64168461:Plus
uniq_2576353	TGTCAAACGGTAACGCAGGTGTCCTAAGGCG	dre_piR_0058848	TGTCAAACGGTAACGCAGGTGTCCTAAGGCG	Danio_rerio:5:64167959:64167989:Plus
uniq_1515257	TGGGAATACCAGGTGCTGTAAGCTTA	dre_piR_0059985	CCGCCTGGGAATACCAGGTGCTGTAAGCTTA	Danio_rerio:7:1187692:1187722:Minus
uniq_2355707	CCTGGGAATACCAGGTGCTGTAAGCTTA	dre_piR_0059987	CCTGGGAATACCAGGTGCTGTAAGCTTA	Danio_rerio:7:1187692:1187719:Minus
uniq_66021	CAGAAGGGCAAAAGCTCGCTTGATCT	dre_piR_0061523	CAGAAGGGCAAAAGCTCGCTTGATCT	Danio_rerio:5:64168021:64168046:Plus
uniq_231639	TTGGTGAATTCTGCTTCACAATGATA	dre_piR_0064967	TTGGTGAATTCTGCTTCACAATGATA	Danio_rerio:5:64168214:64168239:Minus
uniq_1979803	AAGGGAACGGGCTTGGCAGAATCAGC	dre_piR_0065052	AAGGGAACGGGCTTGGCAGAATCAGC	Danio_rerio:5:64167652:64167677:Plus
uniq_1947476	GCATTGGTGGTTCAGTGGTAGAGTTC	dre_piR_0065738	GCATTGGTGGTTCAGTGGTAGAGTTC	Danio_rerio:14:29865313:29865338:Minus
uniq_58521	AGCAGTTGAACATGGGTCAGTCGGTC	hsa_piR_000753	AGCAGTTGAACATGGGTCAGTCGGTCCTG	gb|DQ570940|Homo
uniq_707672	AGCAGTTGAACATGGGTCAGTCGGT	hsa_piR_000753	AGCAGTTGAACATGGGTCAGTCGGTCCTG	gb|DQ570940|Homo
uniq_785489	AGCAGTTGAACATGGGTCAGTCGGTCC	hsa_piR_000753	AGCAGTTGAACATGGGTCAGTCGGTCCTG	gb|DQ570940|Homo
uniq_805840	GCAGTTGAACATGGGTCAGTCGGTC	hsa_piR_000753	AGCAGTTGAACATGGGTCAGTCGGTCCTG	gb|DQ570940|Homo
uniq_832647	AGCAGTTGAACATGGGTCAGTCGGTCCT	hsa_piR_000753	AGCAGTTGAACATGGGTCAGTCGGTCCTG	gb|DQ570940|Homo
uniq_1321030	GCAGTTGAACATGGGTCAGTCGGTCCT	hsa_piR_000753	AGCAGTTGAACATGGGTCAGTCGGTCCTG	gb|DQ570940|Homo
uniq_2069273	CAGTTGAACATGGGTCAGTCGGTCC	hsa_piR_000753	AGCAGTTGAACATGGGTCAGTCGGTCCTG	gb|DQ570940|Homo
uniq_2606757	GCAGTTGAACATGGGTCAGTCGGTCC	hsa_piR_000753	AGCAGTTGAACATGGGTCAGTCGGTCCTG	gb|DQ570940|Homo
uniq_2868282	AGCATTGGTGGTTCAGTGGTAGAATTCT	hsa_piR_000765	AGCATTGGTGGTTCAGTGGTAGAATTCTCGC	gb|DQ570956|Homo
uniq_921388	TTGGTGGTTCAGTGGTAGAATTCTCGC	hsa_piR_001311	ATTGGTGGTTCAGTGGTAGAATTCTCGCC	gb|DQ571812|Homo
uniq_460176	TTGGTGGTTCAGTGGTAGAATTCTCGCCTG	hsa_piR_001312	ATTGGTGGTTCAGTGGTAGAATTCTCGCCTG	gb|DQ571813|Homo
uniq_4440	ATTTGGTGTATGTGCTTGGCTGAGGAG	hsa_piR_001318	ATTTGGTGTATGTGCTTGGCTGAGGAGCCAA	gb|DQ571823|Homo
uniq_712635	ATTTGGTGTATGTGCTTGGCTGAGGA	hsa_piR_001318	ATTTGGTGTATGTGCTTGGCTGAGGAGCCAA	gb|DQ571823|Homo
uniq_855423	TTGGTGTATGTGCTTGGCTGAGGAGC	hsa_piR_001318	ATTTGGTGTATGTGCTTGGCTGAGGAGCCAA	gb|DQ571823|Homo
uniq_873648	ATTTGGTGTATGTGCTTGGCTGAGG	hsa_piR_001318	ATTTGGTGTATGTGCTTGGCTGAGGAGCCAA	gb|DQ571823|Homo
uniq_1028233	ATTTGGTGTATGTGCTTGGCTGAGGAGC	hsa_piR_001318	ATTTGGTGTATGTGCTTGGCTGAGGAGCCAA	gb|DQ571823|Homo
uniq_1319892	TTTGGTGTATGTGCTTGGCTGAGGAGC	hsa_piR_001318	ATTTGGTGTATGTGCTTGGCTGAGGAGCCAA	gb|DQ571823|Homo
uniq_2165327	ATTTGGTGTATGTGCTTGGCTGAGGAGCCA	hsa_piR_001318	ATTTGGTGTATGTGCTTGGCTGAGGAGCCAA	gb|DQ571823|Homo
uniq_2230646	ATTTGGTGTATGTGCTTGGCTGAGGAGCCAA	hsa_piR_001318	ATTTGGTGTATGTGCTTGGCTGAGGAGCCAA	gb|DQ571823|Homo
uniq_1911329	TGAAGGTGTTCCTGGAGAACGTGATT	hsa_piR_006658	TGAAGGTGTTCCTGGAGAACGTGATTC	gb|DQ579110|Homo
uniq_777048	CAGTTGAACATGGGTCAGTCGGTCCT	hsa_piR_009228	CAGTTGAACATGGGTCAGTCGGTCCT	gb|DQ582496|Homo
uniq_683343	TGTATGTGCTTGGCTGAGGAGCCAAT	hsa_piR_014923	TGGTGTATGTGCTTGGCTGAGGAGCCAATGG	gb|DQ590404|Homo
uniq_450946	TGGTTCAGTGGTAGAATTCTCGCCTGC	hsa_piR_018570	TTGGTGGTTCAGTGGTAGAATTCTCGCCTGCC	gb|DQ595536|Homo
uniq_562695	TTGGTGGTTCAGTGGTAGAATTCTCGCCTGC	hsa_piR_018570	TTGGTGGTTCAGTGGTAGAATTCTCGCCTGCC	gb|DQ595536|Homo
uniq_1134381	GTGGTTCAGTGGTAGAATTCTCGCCTGCC	hsa_piR_018570	TTGGTGGTTCAGTGGTAGAATTCTCGCCTGCC	gb|DQ595536|Homo
uniq_1865339	GGTTCAGTGGTAGAATTCTCGCCTGC	hsa_piR_018570	TTGGTGGTTCAGTGGTAGAATTCTCGCCTGCC	gb|DQ595536|Homo
uniq_531703	TTGGTGGTTCAGTGGTAGAATTCTCGCCTGCC	hsa_piR_018570	TTGGTGGTTCAGTGGTAGAATTCTCGCCTGCC	gb|DQ595536|Homo

uniq_556109	TGGTTCAGTGGTAGAATTCTCGCCTGCC	hsa_piR_018570	TTGGTGGTTCAGTGGTAGAATTCTCGCCTGCC	gb|DQ595536|Homo
uniq_7740	TTGGTGTATGTGCTTGGCTGAGGAGCC	hsa_piR_018573	TTGGTGTATGTGCTTGGCTGAGGAGCC	gb|DQ595539|Homo
uniq_2761330	TGGTGTATGTGCTTGGCTGAGGAGC	hsa_piR_018573	TTGGTGTATGTGCTTGGCTGAGGAGCC	gb|DQ595539|Homo
uniq_66454	GCATTGGTGGTTCAGTGGTAGAATTCTCA	hsa_piR_019825	GCATTGGTGGTTCAGTGGTAGAATTCTCAC	gb|DQ597218|Homo
uniq_183491	GCATTGGTGGTTCAGTGGTAGAATTCTCAC	hsa_piR_019825	GCATTGGTGGTTCAGTGGTAGAATTCTCAC	gb|DQ597218|Homo
uniq_860961	GGGGATGTAGCTCAGTGGTAGAGCGCA	hsa_piR_020485	GGGGATGTAGCTCAGTGGTAGAGCGCATGCT	gb|DQ598159|Homo
uniq_446107	GGGGATGTAGCTCAGTGGTAGAGCGC	hsa_piR_020485	GGGGATGTAGCTCAGTGGTAGAGCGCATGCT	gb|DQ598159|Homo
uniq_642070	GGTGTTGACGCGATGTGATTTCTGCC	mmu_piR_000622	CGGGTGTTGACGCGATGTGATTTCTGCC	gb|DQ540988|Mus_musculus:3:5843428:5843455:Plus
uniq_201553	GGGTGTTGACGCGATGTGATTTCTGCC	mmu_piR_000622	CGGGTGTTGACGCGATGTGATTTCTGCC	gb|DQ540988|Mus_musculus:3:5843428:5843455:Plus
uniq_952674	CGAAAGATGGTGAACTATGCCTGGGC	mmu_piR_018323	CGAAAGATGGTGAACTATGCCTGGGC	gb|DQ698371|Mus_musculus:15:86022679:86022704:Plus
uniq_2246269	TCAGACCCCAGAAAAGGTGTTGGTTGAT	mmu_piR_038323	ATCAGACCCCAGAAAAGGTGTTGGTTGATAT	gb|piRNA-T34|Mus_musculus:13:44880547:44880577:Minus
uniq_965909	GCATGGGTGGTTCAGTGGTAGAATTCTCGCC	rno_piR_005567	GCATGGGTGGTTCAGTGGTAGAATTCTCGCC	gb|DQ614031|Rattus
uniq_73519	GCATGGGTGGTTCAGTGGTAGAATTCTC	rno_piR_005567	GCATGGGTGGTTCAGTGGTAGAATTCTCGCC	gb|DQ614031|Rattus
uniq_1569095	GCATGGGTGGTTCAGTGGTAGAATTCT	rno_piR_005567	GCATGGGTGGTTCAGTGGTAGAATTCTCGCC	gb|DQ614031|Rattus
uniq_2757252	GCATGGGTGGTTCAGTGGTAGAATTCTCG	rno_piR_005567	GCATGGGTGGTTCAGTGGTAGAATTCTCGCC	gb|DQ614031|Rattus
uniq_1603788	TTGACGCGATGTGATTTCTGCCCAGT	rno_piR_005787	GGGTGTTGACGCGATGTGATTTCTGCCCAGT	gb|DQ614419|Rattus
uniq_2330815	TGACGCGATGTGATTTCTGCCCAGT	rno_piR_005787	GGGTGTTGACGCGATGTGATTTCTGCCCAGT	gb|DQ614419|Rattus

Table S3 - Summary of 265 enriched GO annotations and 29 enriched KEGG pathways that may associate with sturgeon putative piRNA.

Enriched GO accession	Description	Term type	Over-represented pValue	Corrected pValue
GO:0015074	DNA integration	biological_process	3.01E-171	2.00E-167
GO:0006313	transposition, DNA-mediated	biological_process	3.67E-50	8.10E-47
GO:0032196	transposition	biological_process	3.67E-50	8.10E-47
GO:0006310	DNA recombination	biological_process	1.77E-32	2.93E-29
GO:0006259	DNA metabolic process	biological_process	2.63E-29	3.48E-26
GO:0034641	cellular nitrogen compound metabolic process	biological_process	1.58E-28	1.75E-25
GO:0071704	organic substance metabolic process	biological_process	1.53E-23	1.26E-20
GO:0044238	primary metabolic process	biological_process	8.25E-23	6.08E-20
GO:0006807	nitrogen compound metabolic process	biological_process	1.22E-22	8.09E-20
GO:0046483	heterocycle metabolic process	biological_process	4.42E-20	2.66E-17
GO:0022613	ribonucleoprotein complex biogenesis	biological_process	4.93E-20	2.72E-17
GO:0042254	ribosome biogenesis	biological_process	4.53E-19	2.31E-16
GO:0006139	nucleobase-containing compound metabolic process	biological_process	1.21E-18	5.73E-16
GO:0044237	cellular metabolic process	biological_process	1.35E-18	5.95E-16
GO:0008152	metabolic process	biological_process	1.44E-18	5.97E-16
GO:1901135	carbohydrate derivative metabolic process	biological_process	1.77E-18	6.89E-16
GO:0006725	cellular aromatic compound metabolic process	biological_process	2.29E-18	8.43E-16
GO:1901360	organic cyclic compound metabolic process	biological_process	1.20E-17	4.18E-15
GO:0044710	single-organism metabolic process	biological_process	1.61E-17	5.32E-15
GO:1901564	organonitrogen compound metabolic process	biological_process	9.98E-17	3.15E-14
GO:0055086	nucleobase-containing small molecule metabolic process	biological_process	6.28E-16	1.89E-13
GO:0009116	nucleoside metabolic process	biological_process	1.64E-15	4.73E-13
GO:1901657	glycosyl compound metabolic process	biological_process	2.15E-15	5.93E-13
GO:1901566	organonitrogen compound biosynthetic process	biological_process	3.49E-15	9.25E-13
GO:0072527	pyrimidine-containing compound metabolic process	biological_process	5.29E-15	1.30E-12
GO:0042278	purine nucleoside metabolic process	biological_process	7.36E-15	1.63E-12
GO:0046128	purine ribonucleoside metabolic process	biological_process	7.36E-15	1.63E-12
GO:0009141	nucleoside triphosphate metabolic process	biological_process	3.27E-14	6.98E-12
GO:0043170	macromolecule metabolic process	biological_process	4.98E-14	1.03E-11

GO:0009123	nucleoside monophosphate metabolic process	biological_process	7.48E-14	1.50E-11
GO:0009119	ribonucleoside metabolic process	biological_process	7.71E-14	1.50E-11
GO:0009199	ribonucleoside triphosphate metabolic process	biological_process	1.39E-13	2.56E-11
GO:0009205	purine ribonucleoside triphosphate metabolic process	biological_process	1.39E-13	2.56E-11
GO:0072521	purine-containing compound metabolic process	biological_process	1.61E-13	2.87E-11
GO:0009144	purine nucleoside triphosphate metabolic process	biological_process	1.70E-13	2.97E-11
GO:0006753	nucleoside phosphate metabolic process	biological_process	3.31E-13	5.62E-11
GO:0046034	ATP metabolic process	biological_process	5.35E-13	8.86E-11
GO:0009161	ribonucleoside monophosphate metabolic process	biological_process	5.78E-13	9.33E-11
GO:0009117	nucleotide metabolic process	biological_process	7.96E-13	1.23E-10
GO:0009126	purine nucleoside monophosphate metabolic process	biological_process	9.14E-13	1.35E-10
GO:0009167	purine ribonucleoside monophosphate metabolic process	biological_process	9.14E-13	1.35E-10
GO:0006518	peptide metabolic process	biological_process	2.15E-12	3.10E-10
GO:0043603	cellular amide metabolic process	biological_process	3.85E-12	5.42E-10
GO:0006412	translation	biological_process	3.94E-12	5.42E-10
GO:0090304	nucleic acid metabolic process	biological_process	4.72E-12	6.25E-10
GO:0009259	ribonucleotide metabolic process	biological_process	5.83E-12	7.57E-10
GO:0006206	pyrimidine nucleobase metabolic process	biological_process	6.78E-12	8.64E-10
GO:0044711	single-organism biosynthetic process	biological_process	7.40E-12	9.25E-10
GO:0009150	purine ribonucleotide metabolic process	biological_process	8.10E-12	9.94E-10
GO:0044260	cellular macromolecule metabolic process	biological_process	1.22E-11	1.47E-09
GO:0043043	peptide biosynthetic process	biological_process	1.60E-11	1.89E-09
GO:0006163	purine nucleotide metabolic process	biological_process	1.84E-11	2.14E-09
GO:0019637	organophosphate metabolic process	biological_process	1.87E-11	2.14E-09
GO:0019693	ribose phosphate metabolic process	biological_process	2.01E-11	2.26E-09
GO:0009058	biosynthetic process	biological_process	2.06E-11	2.28E-09
GO:1901576	organic substance biosynthetic process	biological_process	2.79E-11	3.03E-09
GO:0043604	amide biosynthetic process	biological_process	2.47E-10	2.48E-08
GO:0044249	cellular biosynthetic process	biological_process	5.24E-10	5.04E-08
GO:0006118	obsolete electron transport	biological_process	4.58E-09	4.27E-07
GO:0055114	oxidation-reduction process	biological_process	6.84E-09	6.12E-07
GO:0044281	small molecule metabolic process	biological_process	1.54E-08	1.34E-06
GO:0006352	DNA-templated transcription, initiation	biological_process	2.50E-08	2.15E-06
GO:0072528	pyrimidine-containing compound biosynthetic process	biological_process	2.92E-08	2.48E-06
GO:0042775	mitochondrial ATP synthesis coupled electron transport	biological_process	4.57E-08	3.83E-06

GO:0006091	generation of precursor metabolites and energy	biological_process	5.05E-08	4.19E-06
GO:0006119	oxidative phosphorylation	biological_process	5.72E-08	4.68E-06
GO:0045333	cellular respiration	biological_process	7.14E-08	5.77E-06
GO:0006733	oxidoreduction coenzyme metabolic process	biological_process	9.60E-08	7.67E-06
GO:0006732	coenzyme metabolic process	biological_process	1.37E-07	1.07E-05
GO:0044283	small molecule biosynthetic process	biological_process	1.56E-07	1.16E-05
GO:0015980	energy derivation by oxidation of organic compounds	biological_process	1.73E-07	1.28E-05
GO:0042773	ATP synthesis coupled electron transport	biological_process	2.86E-07	2.08E-05
GO:0070647	protein modification by small protein conjugation or removal	biological_process	8.94E-07	6.17E-05
GO:0010467	gene expression	biological_process	1.13E-06	7.66E-05
GO:0044271	cellular nitrogen compound biosynthetic process	biological_process	1.29E-06	8.54E-05
GO:0016567	protein ubiquitination	biological_process	1.78E-06	0.00011541
GO:0006220	pyrimidine nucleotide metabolic process	biological_process	1.92E-06	0.00012335
GO:0022904	respiratory electron transport chain	biological_process	2.14E-06	0.00013362
GO:0032446	protein modification by small protein conjugation	biological_process	2.70E-06	0.00016654
GO:0006120	mitochondrial electron transport, NADH to ubiquinone	biological_process	2.71E-06	0.00016654
GO:0006743	ubiquinone metabolic process	biological_process	5.58E-06	0.00032981
GO:0006744	ubiquinone biosynthetic process	biological_process	5.58E-06	0.00032981
GO:0009064	glutamine family amino acid metabolic process	biological_process	8.58E-06	0.00048603
GO:0019915	lipid storage	biological_process	9.86E-06	0.0005536
GO:0033057*	multicellular organismal reproductive behavior	biological_process	1.14E-05	0.00061935
GO:0045924*	regulation of female receptivity	biological_process	1.14E-05	0.00061935
GO:0046008*	regulation of female receptivity, post-mating	biological_process	1.14E-05	0.00061935
GO:0060180*	female mating behavior	biological_process	1.14E-05	0.00061935
GO:0006818	hydrogen transport	biological_process	1.26E-05	0.00067398
GO:0015992	proton transport	biological_process	1.26E-05	0.00067398
GO:0022900	electron transport chain	biological_process	1.30E-05	0.00068736
GO:0009112	nucleobase metabolic process	biological_process	1.62E-05	0.00084947
GO:0006165	nucleoside diphosphate phosphorylation	biological_process	1.68E-05	0.00086969
GO:0046939	nucleotide phosphorylation	biological_process	1.68E-05	0.00086969
GO:0006814	sodium ion transport	biological_process	1.91E-05	0.00098201
GO:0006221	pyrimidine nucleotide biosynthetic process	biological_process	1.97E-05	0.0010037
GO:0009132	nucleoside diphosphate metabolic process	biological_process	2.79E-05	0.0013906
GO:0042181	ketone biosynthetic process	biological_process	3.34E-05	0.0016288
GO:1901661	quinone metabolic process	biological_process	3.34E-05	0.0016288

GO:1901663	quinone biosynthetic process	biological_process	3.34E-05	0.0016288
GO:0007618*	mating	biological_process	3.58E-05	0.0017297
GO:0090407	organophosphate biosynthetic process	biological_process	5.01E-05	0.002371
GO:0045454	cell redox homeostasis	biological_process	5.53E-05	0.0025961
GO:0007617*	mating behavior	biological_process	5.93E-05	0.0026927
GO:0019098*	reproductive behavior	biological_process	5.93E-05	0.0026927
GO:0044705*	multi-organism reproductive behavior	biological_process	5.93E-05	0.0026927
GO:1901137	carbohydrate derivative biosynthetic process	biological_process	8.77E-05	0.0039267
GO:0051186	cofactor metabolic process	biological_process	9.65E-05	0.0042893
GO:0009108	coenzyme biosynthetic process	biological_process	9.81E-05	0.004333
GO:0042180	cellular ketone metabolic process	biological_process	0.00012845	0.0054551
GO:0010883	regulation of lipid storage	biological_process	0.00013348	0.005597
GO:0010884	positive regulation of lipid storage	biological_process	0.00013348	0.005597
GO:0009059	macromolecule biosynthetic process	biological_process	0.00015638	0.00645
GO:0006006	glucose metabolic process	biological_process	0.00016012	0.0064681
GO:0044706	multi-multicellular organism process	biological_process	0.00018404	0.0073893
GO:0006144	purine nucleobase metabolic process	biological_process	0.00019364	0.0076362
GO:0048609*	multicellular organismal reproductive process	biological_process	0.00020382	0.007943
GO:0002200	somatic diversification of immune receptors	biological_process	0.0002182	0.0083052
GO:0002562	somatic diversification of immune receptors via germline recombination within a single locus	biological_process	0.0002182	0.0083052
GO:0016444	somatic cell DNA recombination	biological_process	0.0002182	0.0083052
GO:0033151	V(D)J recombination	biological_process	0.0002182	0.0083052
GO:0034645	cellular macromolecule biosynthetic process	biological_process	0.00027445	0.010236
GO:0015947	methane metabolic process	biological_process	0.00027656	0.010236
GO:0043446	cellular alkane metabolic process	biological_process	0.00027656	0.010236
GO:0009229	thiamine diphosphate biosynthetic process	biological_process	0.00031778	0.011632
GO:0042357	thiamine diphosphate metabolic process	biological_process	0.00031778	0.011632
GO:0006096	glycolytic process	biological_process	0.0003288	0.011839
GO:0006757	ATP generation from ADP	biological_process	0.0003288	0.011839
GO:0046031	ADP metabolic process	biological_process	0.0003288	0.011839
GO:0006974	cellular response to DNA damage stimulus	biological_process	0.00035712	0.012789
GO:0042435	indole-containing compound biosynthetic process	biological_process	0.00036713	0.013014
GO:0034470	ncRNA processing	biological_process	0.00036929	0.013014
GO:0006094	gluconeogenesis	biological_process	0.0004126	0.014237

GO:0019319	hexose biosynthetic process	biological_process	0.0004126	0.014237
GO:0046364	monosaccharide biosynthetic process	biological_process	0.0004126	0.014237
GO:0006541	glutamine metabolic process	biological_process	0.00045278	0.015452
GO:0009135	purine nucleoside diphosphate metabolic process	biological_process	0.00047319	0.015833
GO:0009179	purine ribonucleoside diphosphate metabolic process	biological_process	0.00047319	0.015833
GO:0044724	single-organism carbohydrate catabolic process	biological_process	0.00052945	0.017451
GO:0009185	ribonucleoside diphosphate metabolic process	biological_process	0.00060607	0.019682
GO:0042430	indole-containing compound metabolic process	biological_process	0.00062269	0.019929
GO:0009147	pyrimidine nucleoside triphosphate metabolic process	biological_process	0.00062951	0.020006
GO:0042451	purine nucleoside biosynthetic process	biological_process	0.0007396	0.023004
GO:0046129	purine ribonucleoside biosynthetic process	biological_process	0.0007396	0.023004
GO:0006629	lipid metabolic process	biological_process	0.00075402	0.023272
GO:1901607	alpha-amino acid biosynthetic process	biological_process	0.00075523	0.023272
GO:0000380	alternative mRNA splicing, via spliceosome	biological_process	0.00075981	0.023304
GO:0022414*	reproductive process	biological_process	0.00077751	0.023628
GO:0008610	lipid biosynthetic process	biological_process	0.00078755	0.023824
GO:0001819	positive regulation of cytokine production	biological_process	0.00079315	0.023885
GO:0009142	nucleoside triphosphate biosynthetic process	biological_process	0.0008372	0.024542
GO:0009145	purine nucleoside triphosphate biosynthetic process	biological_process	0.0008372	0.024542
GO:0009201	ribonucleoside triphosphate biosynthetic process	biological_process	0.0008372	0.024542
GO:0009206	purine ribonucleoside triphosphate biosynthetic process	biological_process	0.0008372	0.024542
GO:0051705	multi-organism behavior	biological_process	0.0010251	0.029786
GO:0008652	cellular amino acid biosynthetic process	biological_process	0.0010565	0.030409
GO:0046500	S-adenosylmethionine metabolic process	biological_process	0.0010854	0.030995
GO:0006825	copper ion transport	biological_process	0.0011031	0.031365
GO:0019953*	sexual reproduction	biological_process	0.001153	0.032644
GO:0044272	sulfur compound biosynthetic process	biological_process	0.0013301	0.037182
GO:0007610	behavior	biological_process	0.0014508	0.039882
GO:0019538	protein metabolic process	biological_process	0.0014662	0.04014
GO:0034660	ncRNA metabolic process	biological_process	0.0014957	0.040778
GO:0033554*	cellular response to stress	biological_process	0.0015323	0.041604
GO:0006487	protein N-linked glycosylation	biological_process	0.0015724	0.04252
GO:0019362	pyridine nucleotide metabolic process	biological_process	0.0016549	0.044317
GO:0009309	amine biosynthetic process	biological_process	0.001659	0.044317
GO:0042401	cellular biogenic amine biosynthetic process	biological_process	0.001659	0.044317

GO:0044703*	multi-organism reproductive process	biological_process	0.0017665	0.046625
GO:0006525	arginine metabolic process	biological_process	0.0018271	0.047572
GO:0001816	cytokine production	biological_process	0.0018383	0.047572
GO:0001817	regulation of cytokine production	biological_process	0.0018383	0.047572
GO:0006123	mitochondrial electron transport, cytochrome c to oxygen	biological_process	0.0019811	0.049862
GO:0072524	pyridine-containing compound metabolic process	biological_process	0.001987	0.049862
GO:0000150	recombinase activity	molecular_function	6.33E-24	5.99E-21
GO:0003735	structural constituent of ribosome	molecular_function	4.21E-15	1.07E-12
GO:0003676	nucleic acid binding	molecular_function	5.86E-15	1.39E-12
GO:0004842	ubiquitin-protein transferase activity	molecular_function	5.04E-11	5.30E-09
GO:0019787	ubiquitin-like protein transferase activity	molecular_function	5.04E-11	5.30E-09
GO:0016491	oxidoreductase activity	molecular_function	1.22E-10	1.26E-08
GO:0004803	transposase activity	molecular_function	2.61E-10	2.58E-08
GO:0046914	transition metal ion binding	molecular_function	3.43E-10	3.35E-08
GO:0008270	zinc ion binding	molecular_function	5.19E-09	4.78E-07
GO:0034062	RNA polymerase activity	molecular_function	5.89E-09	5.34E-07
GO:0003899	DNA-directed RNA polymerase activity	molecular_function	1.44E-08	1.27E-06
GO:0000990	transcription factor activity, core RNA polymerase binding	molecular_function	1.42E-07	1.07E-05
GO:0000996	core DNA-dependent RNA polymerase binding promoter specificity activity	molecular_function	1.42E-07	1.07E-05
GO:0016987	sigma factor activity	molecular_function	1.42E-07	1.07E-05
GO:0016651	oxidoreductase activity, acting on NAD(P)H	molecular_function	4.72E-07	3.33E-05
GO:0003954	NADH dehydrogenase activity	molecular_function	1.06E-06	7.27E-05
GO:0016655	oxidoreductase activity, acting on NAD(P)H, quinone or similar compound as acceptor	molecular_function	1.37E-06	8.96E-05
GO:0008137	NADH dehydrogenase (ubiquinone) activity	molecular_function	1.99E-06	0.00012586
GO:0050136	NADH dehydrogenase (quinone) activity	molecular_function	1.99E-06	0.00012586
GO:0016860	intramolecular oxidoreductase activity	molecular_function	7.23E-06	0.00042391
GO:0004523	RNA-DNA hybrid ribonuclease activity	molecular_function	7.48E-06	0.00043459
GO:0009055	electron carrier activity	molecular_function	4.03E-05	0.0019333
GO:0003824	catalytic activity	molecular_function	4.43E-05	0.0021105
GO:0016779	nucleotidyltransferase activity	molecular_function	5.75E-05	0.002682
GO:0000988	transcription factor activity, protein binding	molecular_function	0.00010908	0.0047543
GO:0030975	thiamine binding	molecular_function	0.00015119	0.0062996
GO:0008234	cysteine-type peptidase activity	molecular_function	0.00015675	0.00645
GO:0004298	threonine-type endopeptidase activity	molecular_function	0.00015941	0.0064681

GO:0070003	threonine-type peptidase activity	molecular_function	0.00015941	0.0064681
GO:0004576	oligosaccharyl transferase activity	molecular_function	0.00018869	0.0075304
GO:0015078	hydrogen ion transmembrane transporter activity	molecular_function	0.00020245	0.0079364
GO:0016776	phosphotransferase activity, phosphate group as acceptor	molecular_function	0.00023975	0.0090247
GO:0030058	amine dehydrogenase activity	molecular_function	0.00036807	0.013014
GO:0042302	structural constituent of cuticle	molecular_function	0.00045496	0.015452
GO:0016151	nickel cation binding	molecular_function	0.00054346	0.017824
GO:0043169	cation binding	molecular_function	0.00059876	0.019541
GO:0017176	phosphatidylinositol N-acetylglucosaminyltransferase activity	molecular_function	0.00061852	0.019929
GO:0005173	stem cell factor receptor binding	molecular_function	0.00076568	0.023376
GO:0097159	organic cyclic compound binding	molecular_function	0.00081101	0.024202
GO:1901363	heterocyclic compound binding	molecular_function	0.00081101	0.024202
GO:0046872	metal ion binding	molecular_function	0.0008857	0.025849
GO:0061630	ubiquitin protein ligase activity	molecular_function	0.0010603	0.030409
GO:0061659	ubiquitin-like protein ligase activity	molecular_function	0.0010603	0.030409
GO:0005198	structural molecule activity	molecular_function	0.0013187	0.03702
GO:0051536	iron-sulfur cluster binding	molecular_function	0.0014352	0.039617
GO:0051540	metal cluster binding	molecular_function	0.0014352	0.039617
GO:0016530	metallochaperone activity	molecular_function	0.0017478	0.046316
GO:0016531	copper chaperone activity	molecular_function	0.0017478	0.046316
GO:0015002	heme-copper terminal oxidase activity	molecular_function	0.001829	0.047572
GO:0003723	RNA binding	molecular_function	0.0018342	0.047572
GO:0016863	intramolecular oxidoreductase activity, transposing C=C bonds	molecular_function	0.0018504	0.0477
GO:0016894	endonuclease activity, active with either ribo- or deoxyribonucleic acids and producing 3'-phosphomonoesters	molecular_function	0.0018655	0.047902
GO:0004129	cytochrome-c oxidase activity	molecular_function	0.0019811	0.049862
GO:0016675	oxidoreductase activity, acting on a heme group of donors	molecular_function	0.0019811	0.049862
GO:0016676	oxidoreductase activity, acting on a heme group of donors, oxygen as acceptor	molecular_function	0.0019811	0.049862
GO:0005840	ribosome	cellular_component	6.35E-13	1.00E-10
GO:0030529	ribonucleoprotein complex	cellular_component	4.01E-12	5.42E-10
GO:0005730	nucleolus	cellular_component	2.20E-10	2.24E-08
GO:0044444	cytoplasmic part	cellular_component	1.25E-09	1.18E-07
GO:0044432	endoplasmic reticulum part	cellular_component	1.23E-07	9.71E-06
GO:0005789	endoplasmic reticulum membrane	cellular_component	4.50E-07	3.24E-05

GO:0031974	membrane-enclosed lumen	cellular_component	4.68E-07	3.33E-05
GO:0042175	nuclear outer membrane-endoplasmic reticulum membrane network	cellular_component	8.50E-07	5.92E-05
GO:0005737	cytoplasm	cellular_component	1.27E-06	8.48E-05
GO:0031981	nuclear lumen	cellular_component	4.11E-06	0.00024995
GO:0044428	nuclear part	cellular_component	4.69E-06	0.00028271
GO:0043233	organelle lumen	cellular_component	8.36E-06	0.00047724
GO:0070013	intracellular organelle lumen	cellular_component	8.36E-06	0.00047724
GO:0005839	proteasome core complex	cellular_component	2.26E-05	0.0011413
GO:0008250	oligosaccharyltransferase complex	cellular_component	2.34E-05	0.0011742
GO:1990234	transferase complex	cellular_component	5.93E-05	0.0026927
GO:0071013	catalytic step 2 spliceosome	cellular_component	6.30E-05	0.0028397
GO:0031090	organelle membrane	cellular_component	0.00010086	0.004425
GO:0043189	H4/H2A histone acetyltransferase complex	cellular_component	0.00011551	0.0049692
GO:1902562	H4 histone acetyltransferase complex	cellular_component	0.00011551	0.0049692
GO:0000502	proteasome complex	cellular_component	0.00012509	0.0053464
GO:0070069	cytochrome complex	cellular_component	0.00019103	0.0075782
GO:0005783	endoplasmic reticulum	cellular_component	0.00021938	0.0083052
GO:0045261	proton-transporting ATP synthase complex, catalytic core F(1)	cellular_component	0.00040839	0.014237
GO:0005758	mitochondrial intermembrane space	cellular_component	0.00045714	0.015452
GO:0031970	organelle envelope lumen	cellular_component	0.00045714	0.015452
GO:0031300	intrinsic component of organelle membrane	cellular_component	0.00049743	0.016477
GO:0031301	integral component of organelle membrane	cellular_component	0.00049743	0.016477
GO:0042765	GPI-anchor transamidase complex	cellular_component	0.00062153	0.019929
GO:0098803	respiratory chain complex	cellular_component	0.00063113	0.020006
GO:0030176	integral component of endoplasmic reticulum membrane	cellular_component	0.00073851	0.023004
GO:0031227	intrinsic component of endoplasmic reticulum membrane	cellular_component	0.00073851	0.023004
GO:0098588	bounding membrane of organelle	cellular_component	0.001313	0.037014
GO:0031967	organelle envelope	cellular_component	0.0014042	0.039089
GO:0045277	respiratory chain complex IV	cellular_component	0.0019811	0.049862

**Enriched KEGG pathway**	**Description**	**P-Value**	**Corrected P-Value**
ko03010	Ribosome	4.35E-16	1.30E-13
ko00190	Oxidative phosphorylation	3.74E-09	5.59E-07
ko05012*	Parkinson's disease	6.34E-09	6.32E-07

ko05322	Systemic lupus erythematosus	1.27E-07	9.53E-06
ko04141	Protein processing in endoplasmic reticulum	1.16E-05	0.000692643
ko05010	Alzheimer's disease	2.05E-05	0.001013298
ko03050	Proteasome	2.38E-05	0.001013298
ko03008	Ribosome biogenesis in eukaryotes	2.71E-05	0.001013298
ko00020	Citrate cycle (TCA cycle)	4.08E-05	0.001356803
ko03013	RNA transport	5.73E-05	0.001714131
ko00510	N-Glycan biosynthesis	0.000132394	0.003598708
ko03040	Spliceosome	0.00022511	0.005608982
ko04612*	Antigen processing and presentation	0.000317536	0.007303327
ko03060	Protein export	0.000573958	0.011707161
ko04392	Hippo signaling pathway - multiple species	0.000587316	0.011707161
ko04932*	Non-alcoholic fatty liver disease (NAFLD)	0.000729371	0.013071093
ko04145	Phagosome	0.000743173	0.013071093
ko04142	Lysosome	0.001216221	0.019600913
ko00240	Pyrimidine metabolism	0.001245543	0.019600913
ko05130*	Pathogenic Escherichia coli infection	0.001534493	0.022940664
ko00563	Glycosylphosphatidylinositol(GPI)-anchor biosynthesis	0.00172479	0.023823211
ko05310	Asthma	0.001752878	0.023823211
ko04120	Ubiquitin mediated proteolysis	0.003450748	0.044649459
ko03018	RNA degradation	0.003649776	0.044649459
ko05416*	Viral myocarditis	0.003733232	0.044649459
ko00983	Drug metabolism - other enzymes	0.003990382	0.045582732
ko05169*	Epstein-Barr virus infection	0.004282308	0.045582732
ko05330	Allograft rejection	0.004307584	0.045582732
ko00010	Glycolysis / Gluconeogenesis	0.004421068	0.045582732

***: Enriched GO/KEGG terms may involve in reproduction and aquaculture.**

Table S4 - Summary of 130 enriched GO annotations and 2 enriched KEGG pathways that may associate with sex-specific putative piRNA.

Enriched GO accession	Description	Term type	Over-represented pValue	Corrected pValue
GO:0015074	DNA integration	biological_process	5.9564E-90	3.9461E-86
GO:0006259	DNA metabolic process	biological_process	2.3659E-37	7.8371E-34
GO:0006313	transposition, DNA-mediated	biological_process	4.1082E-33	6.8041E-30
GO:0032196	transposition	biological_process	4.1082E-33	6.8041E-30
GO:0090304	nucleic acid metabolic process	biological_process	9.9349E-29	1.3164E-25
GO:0006310	DNA recombination	biological_process	1.1764E-23	9.742E-21
GO:0006139	nucleobase-containing compound metabolic process	biological_process	3.3515E-23	2.4671E-20
GO:0006725	cellular aromatic compound metabolic process	biological_process	5.665E-21	3.753E-18
GO:0046483	heterocycle metabolic process	biological_process	2.0314E-20	1.2234E-17
GO:1901360	organic cyclic compound metabolic process	biological_process	3.1654E-19	1.6132E-16
GO:0034641	cellular nitrogen compound metabolic process	biological_process	1.6349E-17	7.7364E-15
GO:0044260	cellular macromolecule metabolic process	biological_process	1.0795E-16	3.764E-14
GO:0043170	macromolecule metabolic process	biological_process	5.9295E-15	1.7079E-12
GO:0019219	regulation of nucleobase-containing compound metabolic process	biological_process	9.8293E-15	2.7133E-12
GO:0006355	regulation of transcription, DNA-templated	biological_process	1.1774E-14	3.0001E-12
GO:1903506	regulation of nucleic acid-templated transcription	biological_process	1.1774E-14	3.0001E-12
GO:2001141	regulation of RNA biosynthetic process	biological_process	1.2706E-14	3.1178E-12
GO:0051252	regulation of RNA metabolic process	biological_process	1.4687E-14	3.475E-12
GO:2000112	regulation of cellular macromolecule biosynthetic process	biological_process	6.9074E-14	1.578E-11
GO:0010556	regulation of macromolecule biosynthetic process	biological_process	7.4778E-14	1.6237E-11
GO:0031326	regulation of cellular biosynthetic process	biological_process	7.5975E-14	1.6237E-11
GO:0009889	regulation of biosynthetic process	biological_process	8.2933E-14	1.717E-11
GO:0051171	regulation of nitrogen compound metabolic process	biological_process	9.0128E-14	1.8094E-11
GO:0006807	nitrogen compound metabolic process	biological_process	1.1637E-13	2.2675E-11
GO:0006351	transcription, DNA-templated	biological_process	2.4768E-13	4.4348E-11
GO:0097659	nucleic acid-templated transcription	biological_process	2.4768E-13	4.4348E-11
GO:0010468	regulation of gene expression	biological_process	2.6742E-13	4.6622E-11
GO:0080090	regulation of primary metabolic process	biological_process	3.2747E-13	5.5629E-11

GO:0032774	RNA biosynthetic process	biological_process	3.4505E-13	5.715E-11
GO:0060255	regulation of macromolecule metabolic process	biological_process	4.8941E-13	7.9082E-11
GO:0006352	DNA-templated transcription, initiation	biological_process	2.2197E-12	3.5013E-10
GO:0031323	regulation of cellular metabolic process	biological_process	4.4759E-12	6.8959E-10
GO:0016070	RNA metabolic process	biological_process	1.0846E-11	1.633E-09
GO:0044238	primary metabolic process	biological_process	1.9094E-11	2.8111E-09
GO:0034654	nucleobase-containing compound biosynthetic process	biological_process	3.1926E-11	4.5981E-09
GO:0008284	positive regulation of cell proliferation	biological_process	5.1106E-11	7.2038E-09
GO:0010883	regulation of lipid storage	biological_process	8.0205E-11	1.0844E-08
GO:0010884	positive regulation of lipid storage	biological_process	8.0205E-11	1.0844E-08
GO:0042127	regulation of cell proliferation	biological_process	9.9786E-11	1.3222E-08
GO:0019222	regulation of metabolic process	biological_process	5.0553E-10	6.567E-08
GO:0019438	aromatic compound biosynthetic process	biological_process	6.2309E-10	7.9384E-08
GO:0071704	organic substance metabolic process	biological_process	8.1527E-10	1.0191E-07
GO:0046660*	female sex differentiation	biological_process	1.0912E-09	1.3292E-07
GO:0010467	gene expression	biological_process	1.1058E-09	1.3292E-07
GO:0001819	positive regulation of cytokine production	biological_process	1.1235E-09	1.3292E-07
GO:0044237	cellular metabolic process	biological_process	1.3125E-09	1.5255E-07
GO:0018130	heterocycle biosynthetic process	biological_process	1.5314E-09	1.737E-07
GO:0019915	lipid storage	biological_process	1.5469E-09	1.737E-07
GO:0001816	cytokine production	biological_process	1.6869E-09	1.8321E-07
GO:0001817	regulation of cytokine production	biological_process	1.6869E-09	1.8321E-07
GO:0034645	cellular macromolecule biosynthetic process	biological_process	1.9225E-09	2.0543E-07
GO:0009059	macromolecule biosynthetic process	biological_process	2.8751E-09	3.0234E-07
GO:1901362	organic cyclic compound biosynthetic process	biological_process	3.4087E-09	3.5285E-07
GO:0007548*	sex differentiation	biological_process	9.4071E-09	9.588E-07
GO:0022414*	reproductive process	biological_process	1.0036E-08	1.0074E-06
GO:0044271	cellular nitrogen compound biosynthetic process	biological_process	2.6321E-07	0.000025643
GO:0003006*	developmental process involved in reproduction	biological_process	2.8044E-07	0.000026926
GO:0051240	positive regulation of multicellular organismal process	biological_process	3.0061E-07	0.000028399
GO:0044249	cellular biosynthetic process	biological_process	3.0435E-07	0.000028399
GO:1901576	organic substance biosynthetic process	biological_process	5.2502E-07	0.000048309
GO:0009058	biosynthetic process	biological_process	2.9591E-06	0.00026138
GO:0051235	maintenance of location	biological_process	3.1333E-06	0.00027313
GO:0008152	metabolic process	biological_process	4.8208E-06	0.00041478

GO:0048609*	multicellular organismal reproductive process	biological_process	0.000026904	0.0022005
GO:0008283	cell proliferation	biological_process	0.000029569	0.002389
GO:0019953*	sexual reproduction	biological_process	0.000065602	0.0050537
GO:0033057*	multicellular organismal reproductive behavior	biological_process	0.000076352	0.0056204
GO:0045924*	regulation of female receptivity	biological_process	0.000076352	0.0056204
GO:0046008*	regulation of female receptivity, post-mating	biological_process	0.000076352	0.0056204
GO:0060180*	female mating behavior	biological_process	0.000076352	0.0056204
GO:0010876	lipid localization	biological_process	0.000083716	0.0059131
GO:0044703*	multi-organism reproductive process	biological_process	0.000084389	0.0059131
GO:0007339*	binding of sperm to zona pellucida	biological_process	0.000085313	0.0059131
GO:0009988	cell-cell recognition	biological_process	0.000085313	0.0059131
GO:0035036*	sperm-egg recognition	biological_process	0.000085313	0.0059131
GO:0032879	regulation of localization	biological_process	0.0001056	0.0071387
GO:0032504*	multicellular organism reproduction	biological_process	0.00010949	0.0073269
GO:0009253	peptidoglycan catabolic process	biological_process	0.00014936	0.0097971
GO:0008037	cell recognition	biological_process	0.00017013	0.010943
GO:0032501	multicellular organismal process	biological_process	0.000192	0.012231
GO:0007610	behavior	biological_process	0.00020986	0.013241
GO:0044706	multi-multicellular organism process	biological_process	0.00028622	0.017889
GO:0007617*	mating behavior	biological_process	0.0002961	0.017997
GO:0019098*	reproductive behavior	biological_process	0.0002961	0.017997
GO:0044705*	multi-organism reproductive behavior	biological_process	0.0002961	0.017997
GO:0048522	positive regulation of cellular process	biological_process	0.00030863	0.018588
GO:0051239	regulation of multicellular organismal process	biological_process	0.00044101	0.025629
GO:0048518	positive regulation of biological process	biological_process	0.00049265	0.028136
GO:0007618*	mating	biological_process	0.00050449	0.028566
GO:0034755	iron ion transmembrane transport	biological_process	0.00061795	0.033284
GO:0000003*	reproduction	biological_process	0.00067096	0.035848
GO:0051705	multi-organism behavior	biological_process	0.00069986	0.037092
GO:0006397	mRNA processing	biological_process	0.00074343	0.039089
GO:0005667	transcription factor complex	cellular_component	1.7834E-19	9.8456E-17
GO:0043234	protein complex	cellular_component	7.6312E-06	0.00064816
GO:0016592	mediator complex	cellular_component	0.000046159	0.0035976
GO:0032991	macromolecular complex	cellular_component	0.00048251	0.027797
GO:0032865	ERMES complex	cellular_component	0.0005445	0.030061

GO:0044232	organelle membrane contact site	cellular_component	0.0005445	0.030061
GO:0044233	ER-mitochondrion membrane contact site	cellular_component	0.0005445	0.030061
GO:0030904	retromer complex	cellular_component	0.00092602	0.047426
GO:0001071	nucleic acid binding transcription factor activity	molecular_function	1.6629E-24	1.5739E-21
GO:0003700	transcription factor activity, sequence-specific DNA binding	molecular_function	1.6629E-24	1.5739E-21
GO:0003676	nucleic acid binding	molecular_function	2.2209E-17	9.809E-15
GO:0000990	transcription factor activity, core RNA polymerase binding	molecular_function	4.0429E-17	1.488E-14
GO:0000996	core DNA-dependent RNA polymerase binding promoter specificity activity	molecular_function	4.0429E-17	1.488E-14
GO:0016987	sigma factor activity	molecular_function	4.0429E-17	1.488E-14
GO:0000988	transcription factor activity, protein binding	molecular_function	5.9104E-16	1.9578E-13
GO:0008270	zinc ion binding	molecular_function	1.4319E-15	4.5172E-13
GO:0000150	recombinase activity	molecular_function	2.0914E-15	6.2979E-13
GO:0046914	transition metal ion binding	molecular_function	1.3735E-13	2.5999E-11
GO:0003677	DNA binding	molecular_function	5.3374E-08	5.2777E-06
GO:0046872	metal ion binding	molecular_function	0.000001781	0.00016163
GO:0043169	cation binding	molecular_function	2.1589E-06	0.00019328
GO:0097159	organic cyclic compound binding	molecular_function	0.000014807	0.0012262
GO:1901363	heterocyclic compound binding	molecular_function	0.000014807	0.0012262
GO:0001076	transcription factor activity, RNA polymerase II transcription factor binding	molecular_function	0.000034209	0.002698
GO:0001104	RNA polymerase II transcription cofactor activity	molecular_function	0.000034209	0.002698
GO:0003712	transcription cofactor activity	molecular_function	0.000085684	0.0059131
GO:0000989	transcription factor activity, transcription factor binding	molecular_function	0.000090022	0.0061484
GO:0003796	lysozyme activity	molecular_function	0.00014828	0.0097971
GO:0004523	RNA-DNA hybrid ribonuclease activity	molecular_function	0.00016809	0.010918
GO:0003995	acyl-CoA dehydrogenase activity	molecular_function	0.00042335	0.02494
GO:0061630	ubiquitin protein ligase activity	molecular_function	0.00042539	0.02494
GO:0061659	ubiquitin-like protein ligase activity	molecular_function	0.00042539	0.02494
GO:0004842	ubiquitin-protein transferase activity	molecular_function	0.00055631	0.030209
GO:0019787	ubiquitin-like protein transferase activity	molecular_function	0.00055631	0.030209
GO:0016783	sulfurtransferase activity	molecular_function	0.00077379	0.040365
GO:0004803	transposase activity	molecular_function	0.00081919	0.0424
GO:0005173	stem cell factor receptor binding	molecular_function	0.00093061	0.047426

Enriched KEGG pathway	Description	P-Value	Corrected P-Value
ko00534	Glycosaminoglycan biosynthesis - HS/Hep	1.06E-08	3.82E-07
ko00532	Glycosaminoglycan biosynthesis - CS/DS	2.07E-07	3.72E-06

*: Enriched GO/KEGG terms may involve in reproduction and aquaculture.

Table S5 - Validation of the expression of 311 sex-biased putative piRNAs by micrroarray.

Up-regulated piRNAs in testes	piRNA ID	After normalization		Statistical analysis (F vs. M)		Length of piRNA	piRNA seq
		Median_Male	Median_Female	log2Ratio	P-value		
ASY-piR-1029	uniq_2052249	7.046214555	6.483815777	-1.001820436	0.044194439	26	UAUCAAACUGGUAGGCCCUGGUCAGU
ASY-piR-741	uniq_666257	6.79224803	5.8475794	-1.002171099	0.037021376	29	UAUGUCCUCUUUUAGAGCCUGCAGGUGGU
ASY-piR-1312	uniq_573295	7.410097966	6.22881869	-1.008545109	0.025129032	29	UUGGUCUUUGGAUGCAUCUGGUGGGCACA
ASY-piR-218	uniq_819390	5.574908836	4.564784619	-1.010124217	0.004859692	29	UUCCUGAUGUAUGAACUGAUUAAAGCUUC
ASY-piR-849	uniq_10198	9.448632568	8.435323	-1.013309568	0.028664238	28	UAAUGUCAGAACAGUGCAUGCUGGUGCU
ASY-piR-1786	uniq_414440	6.087462841	5.073248982	-1.014213859	0.042775187	26	UGUACUGCUGUAGCCGAUCUGACCAU
ASY-piR-1648	uniq_440736	12.25949809	11.22370008	-1.019870634	4.11E-05	29	UACAGGAAUCUCUGCAGGGUCUGGAGGCG
ASY-piR-1503	uniq_715514	12.71999481	11.57230548	-1.021435773	0.005270714	26	UUCUUAGAGGGACUGGUGGCUCUCAG
ASY-piR-147	uniq_615837	6.942514505	5.920849053	-1.021665452	0.03724273	26	UCUGCGUACAGGAAUCUCUGCAGGGA
ASY-piR-394	uniq_905882	7.903379777	6.759333407	-1.024210554	0.036299574	29	UCUGCGUACAGGAAUCUCUGCAGGGUCUG
ASY-piR-628	uniq_206632	13.15963486	11.83499768	-1.025248705	0.015767096	26	UCUUAGAGGGACUGGUGGCUCUCAGC
ASY-piR-1645	uniq_441539	14.59828517	13.57070563	-1.027579544	0.002493845	27	UGGGUUCGAUCAGCAGGACCGGAGAGC
ASY-piR-942	uniq_469397	10.43549664	9.279223644	-1.029291636	0.001698626	27	UUAGAACUUCUUUAAACAGGGUGUGUG
ASY-piR-227	uniq_131634	7.342815461	6.336878436	-1.029932506	0.016602762	27	UGAGUACGUUGAAAAUGUUGAGACUGC
ASY-piR-1713	uniq_178076	10.06933787	9.038461789	-1.030876085	3.42E-05	27	UACAGGUAUCUCUGCAGGGUCUGGAGU
ASY-piR-1570	uniq_490270	10.55722435	9.434628228	-1.036708286	0.040059604	28	UCACUGUGACUGGAUCUGUGUUGUAUGC
ASY-piR-1449	uniq_565914	7.749310785	6.502500341	-1.037357078	0.040756391	29	UCUGCGUACAGGUAUCUCUGCAGGGUCUA
ASY-piR-812	uniq_514801	10.08192721	9.033423002	-1.038335304	0.047673195	27	UGAUUGCAUUGCUGUAGAACUGUGUGU
ASY-piR-1000	uniq_197248	9.926790153	8.82336724	-1.038882661	0.018409005	27	UCCUGAUCUGUUGGACUGUUCGGGAGU
ASY-piR-1603	uniq_381705	12.17923526	11.12142559	-1.040216917	0.047685	28	UGCGCUGUGCUGUGUUGUGCUCUGUGGU
ASY-piR-1343	uniq_705989	8.41080465	7.120669887	-1.042560462	0.024541161	30	UCUGCGUACAGGUAUCUCUGCAGGGUCUGU
ASY-piR-567	uniq_690594	14.11695308	12.95222556	-1.052575187	0.014609949	29	UUCUUUCCCAUGAACGAGGAAUUCCCAGU
ASY-piR-802	uniq_101638	9.7474937	8.693486957	-1.054006743	0.002729213	27	UAAAGCAUCUCUGGUCUGUCUGAGAGU
ASY-piR-544	uniq_472772	13.23159621	12.28115729	-1.060732258	0.000903716	29	UUUCAGGAUGGACCAGAACGUGGAGGUGU
ASY-piR-1009	uniq_189163	10.45780836	9.406205005	-1.061025212	0.009321026	27	UAGCUGUAUGAGAGGCUGUGGUCUAUU
ASY-piR-1337	uniq_535821	9.599912842	8.537218401	-1.062694442	0.044479264	26	UUUGCAGGAUUUGAAGAUUUGUGUGC
ASY-piR-540	uniq_553449	6.805206455	5.741466986	-1.063739469	0.019718027	27	UCCUGAUGUAUGAACUGAUUAAAGCAU
ASY-piR-1800	uniq_722663	7.204571144	6.241585987	-1.064475703	0.005157236	26	UGGAAACUGGAGACAUUCUUGAGACG
ASY-piR-564	uniq_946093	13.55322929	12.48863517	-1.064594117	0.009050068	26	ACCGAUUGGAUGGUUUAGUGAGGUCU
ASY-piR-379	uniq_323574	9.341148141	8.276512174	-1.064635967	0.036353451	30	UAUUGUUGUAACUUCUGCCAUGAGGGUGGA
ASY-piR-1519	uniq_99033	6.932706887	6.06608919	-1.064910299	0.001810808	30	UCUCGGCCAAGGUGACCAGAGUCUGGGAUC
ASY-piR-547	uniq_1612612	9.117210185	8.17450962	-1.066001244	0.001035413	30	UACUGUUGGACCAUUCCCGGCUGUGGAGGU
ASY-piR-645	uniq_2131639	13.42307213	12.35590164	-1.067170489	0.00018769	31	UUUCAGGAUGGACCAGAACGUGGAGGUGACU

ASY-piR-234	uniq_481939	7.108524457	6.033423002	-1.071612469	0.031609833	26	UCUUCCUUGGUCCUGUAGAGCUUGGA
ASY-piR-142	uniq_695682	8.830779268	7.552028611	-1.076113108	0.038533686	31	UCUGCGUACAGGAAUCUCUGCAGGGUCUGGA
ASY-piR-979	uniq_1789133	12.46147945	11.3777528	-1.083726652	0.01154684	26	UUUCAGGAUGGACCAGAACGUGGAGA
ASY-piR-783	uniq_2007758	13.34810569	12.25949809	-1.088607605	0.000267572	31	UUUCAGGAUGGACCCGAACAUGGAGGUGACC
ASY-piR-233	uniq_360473	8.764318728	7.687667284	-1.090129896	0.019918671	30	UCUGCGUACAGGAAUCUCUGCAGGGUCUGG
ASY-piR-1330	uniq_312861	9.119157288	7.919359948	-1.09046867	0.048832653	27	UUUGCAGGAUUUGAAGAUUUGUGUGCA
ASY-piR-1813	uniq_689842	11.12142559	10.01657618	-1.092598628	0.004067055	29	UGCUCAUGUGUCAGGGCUUGGUAGGCUGC
ASY-piR-1637	uniq_1717673	9.609178738	8.526825235	-1.094843978	0.004276381	29	UAAUUGACGGUGCAGUGAGAGUUUCCUUU
ASY-piR-1304	uniq_360476	8.182394353	7.084808388	-1.097585966	0.031870061	30	UCUGCGUACAGGAAUCUCUGCAGGGUCUGU
ASY-piR-984	uniq_1487787	9.113308077	8.01355531	-1.099752767	0.04359303	27	ACUACUCCAAAGUCCGAGAGCUGGUGU
ASY-piR-1144	uniq_350534	7.016808288	6.037089319	-1.109267212	0.001067661	27	UGGUCUUGUAGAUGGCGAGUCUAGCAA
ASY-piR-1811	uniq_298248	10.09583653	8.984418459	-1.111418075	0.01324668	30	UACAGGUAUCUCUGCAGGGUCUGUAGGCGU
ASY-piR-1286	uniq_231361	14.8623387	13.74917109	-1.113167612	0.008897623	26	UGUUGGUUGAUAUAGACAGCAGGACA
ASY-piR-865	uniq_300756	11.37368083	10.19926336	-1.114186581	0.016011893	28	UUAGAACUUCUUUAAACAGGGUGUGUGC
ASY-piR-1564	uniq_885688	6.828665428	5.886712714	-1.114419911	0.026188651	29	UACGAGUGUACUGUCUGACUUUGAUAGUC
ASY-piR-1074	uniq_59183	10.73541499	9.647758207	-1.117641449	0.000193587	28	UACAGGUAUCUCUGCAGGGUCUGGAGGU
ASY-piR-1865	uniq_338981	10.24317398	9.18404882	-1.120872849	0.00095528	27	UCUGUAAUUCCAGGGAUUGUGGUGAGU
ASY-piR-1305	uniq_902044	7.330916878	6.209453366	-1.121463512	0.003291343	26	UCCGACUGGUGACAAUGGUAGUCCGC
ASY-piR-456	uniq_349350	9.652546247	8.526825235	-1.125721012	0.0066261	27	UCUACUCCCCUCUCACUGUGGUGCUGU
ASY-piR-1625	uniq_826049	14.70643306	13.36365568	-1.131891388	0.000348873	29	UAGGAGUUGUAAGAUAAGGCUGAGACGGC
ASY-piR-606	uniq_565054	6.46760555	5.348728154	-1.132398536	0.002797567	26	UCCUGAUGAACAGUGCCCUGGUGAUC
ASY-piR-1211	uniq_69274	6.199672345	5.523561956	-1.137028249	0.025386495	26	UUCUACCAAAUCUAGUUCGGACAGGU
ASY-piR-291	uniq_168547	11.57230548	10.31891935	-1.137850994	0.022984394	30	UGAGACUGAUUACUGCUGUCUGAGUGUGUU
ASY-piR-1159	uniq_923129	5.705056346	4.564784619	-1.140271727	0.048906595	25	UCCGUCUGCAGGGUGAACCUCUUGC
ASY-piR-1852	uniq_1693483	9.265810362	8.121533517	-1.144276844	0.022278136	26	AAAGCAUCUCUGGUCUGUCUGAGAGU
ASY-piR-889	uniq_204431	12.62411004	11.60933267	-1.154298659	0.001323123	27	UUUGAAAUGUUACCGCGGGAGGCUGGC
ASY-piR-659	uniq_1197941	6.785724906	5.684164178	-1.156368286	0.00095596	25	UGGGCAAGUUGCAGUUCUGAGACAA
ASY-piR-295	uniq_420483	5.115477217	3.857980995	-1.156969346	0.01503692	29	UCCGGAUCGUGCUGACCCUGUCGACAGCU
ASY-piR-1461	uniq_904903	8.763489037	7.614709844	-1.158154537	0.014728483	25	UCUGUGAACAUAGAGCUGAUGUGAC
ASY-piR-1763	uniq_643071	9.094737505	7.977756926	-1.167178878	0.03115636	28	UGAGCGUGGUUCCUGGUGACGUAGAACG
ASY-piR-855	uniq_2413022	9.671835885	8.59058705	-1.167875926	0.041863979	27	UGGGUGUAAAUUGCUGCACAGGUGUGU
ASY-piR-1768	uniq_391515	6.044394119	5.080373416	-1.169925001	0.017412766	26	UGUUGAAAGUCAAUGAACUGUGAAUG
ASY-piR-945	uniq_1872201	14.6783067	13.39063921	-1.170330438	0.000267945	32	UCUAAGUACACACGGGCUGUACAGUGAAACUG
ASY-piR-179	uniq_252270	14.1582415	12.98355776	-1.17468374	0.000249431	30	UCUGAUGUCAGGAGUUGCGGAGGAACGAGU
ASY-piR-1082	uniq_1738602	8.688250309	7.511752654	-1.176497655	0.03721423	27	UAAGGUUCUGGCUUGUGUAGCGAAUGU
ASY-piR-1041	uniq_2349027	7.637832402	6.286942737	-1.183875914	0.015064519	28	UACUGUUGGACCAUUCCCGGCUGUGGAU
ASY-piR-402	uniq_395184	6.309855263	4.754887502	-1.187458631	0.004844041	28	UCCAUAGAUGUUCGAUAGGAUUCAGAUC
ASY-piR-1839	uniq_316583	14.48413058	13.54004729	-1.188152437	0.01311882	26	UGGUGAACUAUGCCUGGGCAGGGCGU
ASY-piR-754	uniq_944850	13.53237622	12.38082178	-1.189883846	0.005252411	26	UUGUUAAAGUGCCAGUAGGCGGAGGC

ASY-piR-465	uniq_849616	8.79658045	7.606096714	-1.191294619	0.031658378	29	UACAAACACAGACGGUUGUUGUGAUGAGU
ASY-piR-1582	uniq_205451	5.910892526	4.709658248	-1.192074279	0.021527753	26	GAGAAUCGUGUGCAAUAGUCUCCACU
ASY-piR-1845	uniq_834403	11.34272288	9.949826711	-1.194353528	0.007952377	28	UUAGAACUUCUUUAAACAGGGUGUGUGU
ASY-piR-1065	uniq_443238	8.233619677	7.14635653	-1.196320736	0.037706265	27	UGUCAGGACGGUGCAUGCUGGUACUCC
ASY-piR-1061	uniq_2341863	8.657616189	7.479780264	-1.196819028	0.010756538	27	UAUUUUCCCAGGAUCAAGGGCUGGUUU
ASY-piR-555	uniq_1007993	9.026984441	7.919359948	-1.198310986	0.000490431	26	UAUUUUCCCAGGAUCAAGGGCUGGUU
ASY-piR-1864	uniq_424025	13.83323601	12.87504536	-1.199529338	0.037642417	29	UGGGAUUAUGACUGAACGCCUCUAAGUCA
ASY-piR-652	uniq_2331133	7.018663844	5.828665428	-1.200161535	0.009531207	27	UGUCAAGAUUGUCCAUACUGGAGGCUG
ASY-piR-1676	uniq_295154	8.879072651	7.675369018	-1.203703634	0.026601648	27	UCGGUCUGGAGAUGUCACUUGGACAGU
ASY-piR-1401	uniq_408607	6.513069582	5.303780748	-1.209288834	0.009982771	25	UCCGACUGGUGACAAUGGUAGUCCG
ASY-piR-874	uniq_102704	10.48683502	9.235216462	-1.21547931	0.000433135	26	UUACAUACUCAGGGCUGGGUAGCUGU
ASY-piR-670	uniq_2096775	6.963859408	5.790076931	-1.216482915	0.031112632	27	UAAUGAAAAUGUACAACUGAGAUGUGU
ASY-piR-1283	uniq_618527	7.286942737	6.186526969	-1.217195217	0.041095747	27	UUGUUUAGGUCUUGCUUCAGUAGAGUC
ASY-piR-595	uniq_1196291	6.8475794	5.62935662	-1.21822278	0.004366266	29	UGAUGUUGGGCUGCACGAUGCCCUGGGCU
ASY-piR-221	uniq_854155	4.709658248	3.95419631	-1.219445137	0.02590274	29	UCCGAGCUUGCCGUUUCUGAACUUUGGCA
ASY-piR-1198	uniq_72781	7.770388596	6.573647187	-1.226068079	0.020117607	28	UCUGCGUACAGGUAUCUCUGCAGGGUCU
ASY-piR-149	uniq_660139	8.002815016	6.894817763	-1.235734091	0.034172985	29	UGACCUUGUUGUCCUUGCUGAAUGAAAGU
ASY-piR-1333	uniq_269500	11.35342254	9.980615636	-1.238603313	0.009917237	26	UGCGUGCGCUGUGCUGUGAAGUGCUG
ASY-piR-321	uniq_763129	10.67581005	9.601151739	-1.240560544	0.019345498	30	UGAGACUGAUUACAGCUGUCUGAGUGUGUC
ASY-piR-1393	uniq_705991	9.491185027	8.347990391	-1.24197279	0.005577858	30	UCUGCGUACAGGUAUCUCUGCAGGGUCUGG
ASY-piR-1322	uniq_689464	14.32708477	13.14749645	-1.245916358	0.004603608	26	UCAGCUAGGAAUAAUGGAAUAGGACU
ASY-piR-847	uniq_409007	12.56188457	11.44553222	-1.25715299	0.010077583	26	UAGAGGUGAAAUUCUUGGACCGGUGU
ASY-piR-598	uniq_362507	14.08558311	12.82641608	-1.259167033	0.000518773	27	UUUCAGGAUGGACCCGAACGUGGAGGU
ASY-piR-1668	uniq_484825	11.50490173	10.19690944	-1.261727744	0.003608853	27	UCUGAUGAAGGUCGGCGUCGGAUGUGU
ASY-piR-568	uniq_790527	11.09759324	9.968666793	-1.26215425	0.006160916	30	UACUGUUGGACCAUUCCCGGCUGUGGAGGC
ASY-piR-1767	uniq_344366	13.48158923	12.43371583	-1.265205832	0.005463106	29	UCCGGUUCUGUUUUGUGGGUUUCCCGAAC
ASY-piR-1525	uniq_303990	7.581200582	6.309855263	-1.271345319	0.001158914	27	UGAGUACGUUGAAAUUGUUGAGACGGU
ASY-piR-472	uniq_416396	7.65224746	6.276124405	-1.272055306	0.016729925	28	UUGAACUUUUGGCUCUGAAAUGGUCAGU
ASY-piR-1850	uniq_255533	12.02571634	10.72827485	-1.27654525	0.007721739	30	UCUUAGAGGGACUGGUGGCUCUCAGCCACA
ASY-piR-840	uniq_1831773	10.32239765	8.945687679	-1.280686649	0.002243177	27	UGACAUAGGACUCUGUGAGUUGGCGGU
ASY-piR-1608	uniq_38091	11.74209887	10.42504102	-1.281813636	0.0206631	30	UGAGACUGAUUACUGCUGUCUGAGUGUGUC
ASY-piR-755	uniq_944848	12.55310927	11.36431708	-1.287893029	0.000117638	26	UUGUUAAAGUGCCAGUAGGCGGAGGU
ASY-piR-982	uniq_597917	9.858240077	8.669475648	-1.288080324	0.000692916	27	UCCUUUGGUCGGUUUCCAGAUGGCGGU
ASY-piR-622	uniq_856037	8.117210185	6.865217746	-1.288544756	0.018728526	29	UACUGUUGGACCAUUCCCGGCUGUGGAGU
ASY-piR-870	uniq_2363048	13.07795617	11.78252051	-1.29543566	0.001234833	26	UAGAGGUGAAAUUCUUGGACCGGUGG
ASY-piR-1054	uniq_243489	13.14749645	11.77848786	-1.301483774	0.000487013	27	UUGUUAAAGUGCCAGUAGGCGGAGGUG
ASY-piR-417	uniq_609107	8.872418378	7.461479447	-1.302839281	0.04917403	28	UUGGAAAGUGUGAGCUCUGGUUCAAGUG
ASY-piR-610	uniq_2139973	14.25898308	12.9659045	-1.312399748	0.002123888	31	UUUCAGGAUGGACCCGAAUGUGGAGGUGACC
ASY-piR-1675	uniq_4118	9.580572641	8.380821784	-1.316435411	0.026763019	25	UGAGCGUGGUUCCUGGUGACGUAGA

ASY-piR-1073	uniq_2085175	14.21963492	12.90250074	-1.317134185	0.001386888	29	UUUCAGGAUGGACCAGAACGUGGAGGUGG
ASY-piR-894	uniq_639000	11.73287404	10.43749201	-1.317673763	0.000377437	28	UAGCAGUGAUGGCUGGGCGGUCUGACGU
ASY-piR-958	uniq_1840388	12.86024638	11.53219356	-1.328052822	2.70E-06	27	UUGUUAAAGUGCCAGUAGGUGGAGGCA
ASY-piR-1601	uniq_301766	6.996238081	5.62935662	-1.340269731	0.040338389	26	UACUCUGGGUUGACGGUCUGAAUAUC
ASY-piR-275	uniq_494549	6.864186145	5.523561956	-1.340624189	0.029515785	28	UGAGUGCAGCAUACUCUAGGUUGACGGU
ASY-piR-1776	uniq_516831	11.35342254	9.986079047	-1.357419908	0.007289783	29	UUAGAACUUCUUUAAACAGGGUGUGUGCU
ASY-piR-589	uniq_1857243	12.31830743	10.95079888	-1.367508548	0.000573828	29	UUUCAGGAUGGACCCGAACAUGGAGGUGU
ASY-piR-270	uniq_274921	8.774237984	7.409390936	-1.377423207	0.006321019	27	UGAAUCAUCGGGUGCUGAAGCGUCAGA
ASY-piR-44	uniq_396347	6.499845887	5.464886049	-1.380555825	0.020033537	27	UAAUUCCGAGCUUGCCGUUUCUGAGCA
ASY-piR-752	uniq_651528	13.8255551	12.44143126	-1.384123841	0.001021593	27	UCUAUUCUGUGGGUGGUGGUGCAUGGC
ASY-piR-1042	uniq_395211	12.99641464	11.60563384	-1.390780799	6.56E-05	29	UGAUGUUGAGACGGCGGAGAAUGGCCAGU
ASY-piR-731	uniq_1857239	12.35590164	10.96229355	-1.393608086	0.000626825	29	UUUCAGGAUGGACCCGAACAUGGAGGUGA
ASY-piR-424	uniq_364394	8.110265791	6.701595261	-1.40867053	0.015164495	28	UUUUAGUGCAAACGGCUGGAUAUGAUGC
ASY-piR-560	uniq_2020443	13.00501043	11.5075469	-1.442124174	0.008706172	26	UGAUUGUGGAUCAUCCGGGGCUGGGG
ASY-piR-75	uniq_120482	5	3.700439718	-1.442518236	0.019887784	26	UCCGAUUCGGUCUGAAAAUACAAACC
ASY-piR-1468	uniq_591247	11.56811447	10.36322219	-1.446681937	0.014494838	29	UCCUUUGAUCGCUCCAAAGUUACUUGGAC
ASY-piR-1660	uniq_958391	8.258566034	6.743712427	-1.453359578	0.005560937	27	AUAUCUGUGAAAUUUACUGAUGAUGUG
ASY-piR-574	uniq_2113368	9.943979914	8.459431619	-1.484548296	0.009106156	29	UACACUCUCAGCAGCCAUCGGGGUCUGGG
ASY-piR-671	uniq_2098014	13.26079705	11.61371274	-1.48738751	0.001266962	29	UUUCAGGAUGGACCCGAAUGUGGAGGUGA
ASY-piR-1071	uniq_2207804	13.51422091	12.02571634	-1.488504569	3.03E-05	26	UUUCAGGAUGGACCCGAAUGUGGAGG
ASY-piR-469	uniq_558376	10.13699111	8.65731845	-1.493486597	0.004460522	28	UCCCGAAAUGAUGGUGAUUGUAGAAGGU
ASY-piR-1325	uniq_685076	5.741466986	4.247927513	-1.493539473	0.006826677	25	UCCGUCUGCAGGGUGAACCUCUUAC
ASY-piR-1270	uniq_696934	13.95625514	12.43371583	-1.51717129	0.005042723	26	UGAUUCAACUCGAACAGCUGGAGGGC
ASY-piR-920	uniq_2020444	12.268542	10.74216906	-1.526372938	0.002539457	26	UGAUUGUGGAUCAUCCGGGGCUGGGA
ASY-piR-988	uniq_1892758	14.0331792	12.49901537	-1.534163837	0.001373807	29	AUUUUCAGGAUGGACCCGAAUGUGGAGGU
ASY-piR-637	uniq_2280712	13.74023753	12.16715676	-1.535672056	0.001265726	27	UGAUUGUGGAUCAUCCGGGGCUGGGGU
ASY-piR-1441	uniq_298490	7.358284895	6.173260714	-1.568207965	0.020712889	28	UAAUGAUCUGCAUGUUUUGUUGAAUAGU
ASY-piR-994	uniq_1666389	12.83335133	11.38563775	-1.581178704	0.000978403	26	UAAUUGUGGAUCAGCCGGGGCUGGGG
ASY-piR-687	uniq_290095	5.303780748	3.700439718	-1.584962501	0.027074218	29	UGCAUUUGGUGAAAAUCUGUUUAUGACUG
ASY-piR-579	uniq_2032628	14.32708477	12.74185317	-1.585231598	3.80E-06	28	UUUCAGGAUGGACCCGAAUGUGGAGGUG
ASY-piR-1150	uniq_142987	7.16155182	5.303780748	-1.586642984	0.022053631	27	UGUGCUGAAUAAAAUGCUGUAUAGGUU
ASY-piR-770	uniq_572119	9.244958393	7.914883386	-1.625742906	0.004126481	26	UUAGAACUUCUUUAAACAGGGUGUGU
ASY-piR-724	uniq_248332	7.28077077	5.741466986	-1.625989769	0.00585617	26	UUUGUAAUUUUUCAGUUUCUGUGAGG
ASY-piR-673	uniq_2360467	13.37223025	11.76563143	-1.628307493	0.000746197	29	UAAUUGUGGAUCAGCCGGGGCUGGGGAGU
ASY-piR-1594	uniq_123564	12.98841842	11.35342254	-1.634995873	0.002666139	29	UAGGAGUUGUAAGAUAAGGCUGAGACGGU
ASY-piR-1453	uniq_627952	5.609794354	3.874469118	-1.638600464	0.035939113	25	UCCUGAUGUAUGAACUGAUUAAAGU
ASY-piR-583	uniq_50192	12.88734756	11.24396733	-1.643380236	0.000542303	27	UGAUUGUGGAUCAUCCGGGGCUGGGGA
ASY-piR-858	uniq_786249	13.603182	11.95679963	-1.646382368	0.002129472	26	UCUAUUCUGUGGGUGGUGGUGCAUGU
ASY-piR-573	uniq_1099307	13.67748108	12.02928723	-1.648193854	0.000953648	27	UUUCAGGAUGGACCCGAAUGUGGAGGU

ASY-piR-581	uniq_597840	11.42870911	9.672425342	-1.652276081	0.000270704	27	UUCUCUGGAUUGUAGUAGGUGUGAGGU
ASY-piR-845	uniq_9583	9.627533884	8.080817528	-1.654174282	0.00250344	26	UACAGAAAUGUAGGCUGGGCAUUAGU
ASY-piR-612	uniq_1911714	10.74216906	9.236811481	-1.669295768	0.017391062	31	UACACUCUCAGCAGCCAUCGGGGUCUGGGCA
ASY-piR-794	uniq_585155	11.22068062	9.666815756	-1.673305615	0.014617535	27	UCCUUUGAUCGCUCCAAAGUUACUUGG
ASY-piR-789	uniq_669560	11.90350531	10.46947224	-1.680911634	0.01282572	27	UUCAACUCGAACAGCUGGAGGGUGGUC
ASY-piR-626	uniq_986456	12.68642757	11.00093895	-1.685488625	0.000308881	27	UAAUUGUGGAUCAGCCGGGGCUGGGGA
ASY-piR-624	uniq_2020441	13.08425476	11.39178061	-1.692474155	0.000354716	26	UGAUUGUGGAUCAUCCGGGGCUGGGU
ASY-piR-793	uniq_2332609	6.912889336	5.235216462	-1.733113269	0.007172954	26	UGACAAUGUUUCGGACAGUUGAAAUU
ASY-piR-638	uniq_1945945	11.13249973	9.375039431	-1.757460298	0.000707698	30	UACACUCUCAGCAGCCAUCGGGGUCUGGGC
ASY-piR-1003	uniq_268947	5.297680549	3.700439718	-1.7589919	0.016863069	27	UACAGUUGUUUUAAACUGUGAGACUUU
ASY-piR-376	uniq_884690	12.49901537	10.70108982	-1.760219389	0.001398891	27	UUGGAAAUACAGCCGUAGACAUGGGGC
ASY-piR-777	uniq_36994	14.19323016	12.24931101	-1.774670925	0.002692004	27	UCUAUUCUGUGGGUGGUGGUGCAUGGU
ASY-piR-576	uniq_490965	13.15429071	11.66073875	-1.804825172	0.000927818	29	UUUCAGGAUGGACCCGAAUGUGGAGGUGU
ASY-piR-1131	uniq_591245	11.92140707	10.16406888	-1.819817335	0.0327572	29	UCCUUUGAUCGCUCCAAAGUUACUUGGAU
ASY-piR-1661	uniq_4590	5.68182404	3.857980995	-1.823843045	0.032892944	25	CUGUAUAAACUGUUCAAUGGUGACU
ASY-piR-586	uniq_743479	12.95222556	11.10841555	-1.843810007	0.008836778	29	UGCUGUUGAACUAGCCUUGGAGAGGGCGU
ASY-piR-631	uniq_1820232	13.11582939	11.2177558	-1.8980736	0.000163483	25	UGCAGAUGGGAACAUGGCUGGCAGU
ASY-piR-621	uniq_2139972	13.15429071	11.54673372	-1.902118087	0.002141446	31	UUUCAGGAUGGACCCGAAUGUGGAGGUGACU
ASY-piR-1795	uniq_1059880	7.410097966	5.431845787	-1.978252179	0.01441268	26	UACUGUUGUUGAUUCUAUGACGAUGU
ASY-piR-838	uniq_946092	13.20480037	11.39803107	-1.987449397	0.000365814	26	ACCGAUUGGAUGGUUUAGUGAGGUCA
ASY-piR-1023	uniq_180371	3.700439718	1.659079432	-2.041360286	0.007717421	26	UAGCUUCAGAUUUAGGAUAUUCAACU
ASY-piR-1078	uniq_244241	5.247927513	3.273018494	-2.078002512	0.006725692	28	UGAGAUUGUCAUAGGAGUCCUUCAGAUC
ASY-piR-796	uniq_550658	6.981091537	5.014950341	-2.084282596	0.035526633	27	UUUCCUGAUAUGCAGACUCUUGACGGG
ASY-piR-1667	uniq_510697	6.257387843	4.169925001	-2.087462841	0.033048722	27	UGUUUGUCGGGUCUCUUCAGAGACGGU
ASY-piR-1548	uniq_46136	13.74023753	10.57947309	-2.106788157	0.011360204	27	UUGGUGUUUUUCUCUGUAUUUGCAGGA
ASY-piR-1367	uniq_534386	7.614709844	5.667702932	-2.125629423	0.015170855	25	UCAGCUCUGAAUGUGGAAAUACAGG
ASY-piR-323	uniq_881210	6.132713922	3.938599455	-2.194114467	0.002889558	29	UGAAGUUUGACUAUGCAGUAAAUCUGUGU
ASY-piR-462	uniq_778029	5.080373416	3.415037499	-2.239071162	0.040175029	27	AACUGGUAGAGGUCCUUGAUUGAAACA
ASY-piR-224	uniq_270947	5.62935662	3.36923381	-2.26012281	0.042606517	28	UGAGCUUGUAAAGGUCCUGAAACAGUGU
ASY-piR-1555	uniq_719684	3.700439718	1.315295612	-2.385144106	0.005075997	25	UCUAGACUGAUGAGCAUCAAUAACA
ASY-piR-1139	uniq_147259	3.772589504	1.315044421	-2.457545083	0.034484453	26	UCUGAUCAUGCUGUAAAUUAUGAAGC
ASY-piR-370	uniq_898371	4.044394119	0.720129867	-2.728738414	0.047212337	30	UAACAUAAUUACUGCCAUCUAUUUUGAACU
ASY-piR-108	uniq_96374	12.570923	10.04621455	-2.759193455	0.011158346	28	UAAUGUACAGAUCACUAAGGCGGGUAGU
ASY-piR-195	uniq_898271	4.448460501	1.580237514	-2.868222987	0.01920801	28	UGAAGUUUGACUAUGCAGUAAAUCUGUG
ASY-piR-437	uniq_526064	10.78108645	8.088346575	-2.877206382	0.01771982	25	UAAUGUACAGAUCACUAAGGCGGGU
ASY-piR-110	uniq_769219	5.222392421	2.543918318	-3.906736716	0.044421583	27	UAAAGACUUAAUCUCUGCAAGGUGUUA
ASY-piR-1255	uniq_730840	4.044394119	0.719794633	-4.140112546	0.000477559	27	UCUGAUCAUGCUGUAAAUUAUGAAGCU

Up-regulated piRNAs in ovaries	piRNA ID	After normalization		Statistical analysis (F vs. M)		Length of piRNA	piRNA seq
		Median_Male	Median_Female	log2Ratio	P-value		
ASY-piR-122	uniq_502621	4.906890596	13.32685077	8.465339655	0.001871969	26	UGGGAAUAUCAAGUGCUGCAGGCACU
ASY-piR-303	uniq_809899	-0.083249726	8.026523443	8.102841167	0.001898777	31	UGAAAGGACAGAUUUACUUCCACUGCAGUGC
ASY-piR-19	uniq_855876	5.357552005	13.35567227	7.998120267	9.84E-05	27	CCUGGGAAUAUCAAGUGCUGCAGGCAC
ASY-piR-4	uniq_332247	5.790076931	13.24019505	7.772753923	0.000399719	25	UGGGAAUAUCAAGUGCUGCAGGCAA
ASY-piR-144	uniq_502613	5.339850003	13.09206888	7.623015886	1.07E-07	26	UGGGAAUAUCAAGUGCUGCAGGCACC
ASY-piR-151	uniq_855875	6.360481336	13.95625514	7.595773805	8.22E-06	27	CCUGGGAAUAUCAAGUGCUGCAGGCAA
ASY-piR-100	uniq_431484	6.321928095	13.79818488	7.552140717	0.000340795	26	UGGGAAUAUCAAGUGCUGCAGGCAUU
ASY-piR-365	uniq_277058	6.675957033	14.56096965	7.550894144	5.38E-06	26	UUGAAUAAGCCUGAUCUCGUCUGAUU
ASY-piR-488	uniq_431489	5.247927513	12.73089465	7.482967133	2.62E-05	26	UGGGAAUAUCAAGUGCUGCAGGCAUA
ASY-piR-1613	uniq_332246	5.136136688	12.95222556	7.465725698	1.01E-05	25	UGGGAAUAUCAAGUGCUGCAGGCAC
ASY-piR-1605	uniq_332251	6.420662048	13.74917109	7.222997921	2.38E-05	25	UGGGAAUAUCAAGUGCUGCAGGCAU
ASY-piR-16	uniq_809907	0.18355396	7.424866117	7.104927652	0.020238546	31	UGAAAGGACAGAUUUACUUCCACUGCAGUGU
ASY-piR-29	uniq_555127	7.046214555	14.12199211	7.075777556	2.13E-05	29	CACCUGGGAAUAUCAAGUGCUGCAGGCAC
ASY-piR-990	uniq_744089	3	9.45532722	7.039156751	0.000168531	27	UCCUUCAGGUAGGACCACUUCGAUGCU
ASY-piR-418	uniq_50195	6.011227255	13.02161621	6.959120288	4.70E-05	29	ACCUGGGAAUAUCAAGUGCUGCAGGCACC
ASY-piR-6	uniq_855873	6.732450113	13.67748108	6.945030968	0.000127695	27	CCUGGGAAUAUCAAGUGCUGCAGGCAU
ASY-piR-15	uniq_555130	7.283860054	14.25898308	6.906667251	8.51E-06	29	CACCUGGGAAUAUCAAGUGCUGCAGGCAU
ASY-piR-7	uniq_784438	7.327178356	14.21963492	6.866051803	1.81E-05	26	CCUGGGAAUAUCAAGUGCUGCAGGCA
ASY-piR-21	uniq_146212	7.668884984	14.31834274	6.649457751	6.49E-06	26	ACCUGGGAAUAUCAAGUGCUGCAGGC
ASY-piR-1	uniq_594685	6.87036472	13.54848078	6.607922469	0.000184234	27	ACCUGGGAAUAUCAAGUGCUGCAGGCA
ASY-piR-70	uniq_417742	7.86727874	14.30452958	6.353244865	6.93E-06	25	CCUGGGAAUAUCAAGUGCUGCAGGC
ASY-piR-184	uniq_146226	7.868308133	14.11695308	6.351326787	7.55E-06	26	ACCUGGGAAUAUCAAGUGCUGCAGGU
ASY-piR-154	uniq_61991	6.241585987	12.69439416	6.340830454	0.00022779	27	ACCUGGGAAUAUCAAGUGCUGCAGACA
ASY-piR-310	uniq_594695	7.647458426	13.94295734	6.295498911	1.32E-05	27	ACCUGGGAAUAUCAAGUGCUGCAGGCU
ASY-piR-52	uniq_819831	7.548179712	13.82009584	6.271916128	0.000101385	28	CACCUGGGAAUAUCAAGUGCUGCAGGCA
ASY-piR-83	uniq_292933	7.880603904	14.00266854	6.122064632	7.11E-06	27	ACCUGGGAAUAUCAAGUGCUGCAGGUA
ASY-piR-667	uniq_413200	3.95419631	10.02040126	6.066204953	0.000248017	26	UCCUUCAGGUAGGACCACUUCGAUGC
ASY-piR-250	uniq_594687	7.35974956	13.54848078	6.004451504	0.000108907	27	ACCUGGGAAUAUCAAGUGCUGCAGGCG
ASY-piR-112	uniq_504868	9.360481336	14.99628958	5.885588784	4.00E-05	29	ACCACCUGGGAAUAUCAAGUGCUGCAGGC
ASY-piR-2	uniq_137209	8.318919345	14.09345883	5.774539486	2.44E-06	29	CCACCUGGGAAUAUCAAGUGCUGCAGGCA
ASY-piR-309	uniq_137208	8.91064273	14.3934128	5.724231786	7.95E-05	29	CCACCUGGGAAUAUCAAGUGCUGCAGGCG
ASY-piR-170	uniq_147764	8.933198855	14.59828517	5.665086319	2.23E-05	27	CACCUGGGAAUAUCAAGUGCUGCAGGC
ASY-piR-14	uniq_208566	7.692324899	13.53237622	5.535343789	3.02E-05	31	ACCACCUGGGAAUAUCAAGUGCUGCAGGCAC
ASY-piR-367	uniq_811621	8.642352599	14.37558251	5.516565471	2.24E-06	29	CCACCUGGGAAUAUCAAGUGCUGCAGGCU

ASY-piR-324	uniq_717045	9.384244122	14.3719581	5.257092667	2.17E-05	27	CCACCUGGGAAUAUCAAGUGCUGCAGG
ASY-piR-284	uniq_436485	5.969626351	11.08214904	5.11252269	4.57E-05	25	UGGGAAUAUCAAGUGCUGUAGGCAC
ASY-piR-80	uniq_341318	9.084586963	13.79818488	4.914529117	3.35E-05	29	CCACCUGGGAAUAUCAAGUGCUGCAGGUA
ASY-piR-204	uniq_304143	3.922832139	8.20701432	4.902686156	0.000873607	31	UGAAAGGACAGAUUUACUUCCACUGCCGUGU
ASY-piR-286	uniq_228223	3.544320516	8.452584453	4.828544544	0.00209855	29	UGAAAGGACAGAUUUACUUCCACUGCAGU
ASY-piR-786	uniq_56345	-0.111457181	4.624490865	4.735948046	0.014889764	26	UGGAUUGUGGACACAGUCUCGACUGU
ASY-piR-20	uniq_152578	10.31628153	14.92802481	4.611743277	0.000124614	31	AGACCACCUGGGAAUAUCAAGUGCUGCAGGC
ASY-piR-123	uniq_332248	8.083922484	12.90704713	4.538453509	0.000172711	25	UGGGAAUAUCAAGUGCUGCAGGCAG
ASY-piR-466	uniq_382758	10.78299821	15.30128183	4.518283624	2.33E-05	31	AGACCACCUGGGAAUAUCAAGUGCUGCAGAC
ASY-piR-72	uniq_844985	10.94275884	15.36512061	4.422361763	9.65E-05	32	GAGACCACCUGGGAAUAUCAAGUGCUGCAGGC
ASY-piR-86	uniq_846975	11.38685046	15.54531142	4.2931388	1.06E-06	31	UGCGGCCACACCACCUUGAAUAAGCCUGAUC
ASY-piR-1410	uniq_957765	-0.13245417	3.95419631	4.176848289	0.002644438	27	UCCAAUGAUUGAAACUCUGGCCAGUUC
ASY-piR-34	uniq_678371	10.19023623	14.1030692	4.142825875	0.000495955	30	GACCACCUGGGAAUAUCAAGUGCUGCAGGU
ASY-piR-1694	uniq_140535	5.431845787	9.422765614	4.055519949	0.035397598	29	UGUCAAUAAAAACUGGGAAGUGAUCCACU
ASY-piR-118	uniq_680423	10.98892133	14.96402493	3.975103598	7.68E-05	31	GACCACCUGGGAAUAUCAAGUGCUGCAGGUA
ASY-piR-433	uniq_379766	11.45284181	15.37814807	3.764435328	1.53E-05	31	GACCACCUGGGAAUAUCAAGUGCUGCAGGAC
ASY-piR-359	uniq_146197	10.68284832	13.99034037	3.739449839	0.001502969	26	ACCUGGGAAUAUCAAGUGCUGCAGGA
ASY-piR-823	uniq_166929	3.285402219	6.886712714	3.601310495	0.001464917	27	UCCACGUAUUGACCAGCCAGUCUGGCU
ASY-piR-1512	uniq_564110	3.142957954	6.743712427	3.600754473	0.046530871	25	UGACUCAGUAGCUACCAUUAAAUGU
ASY-piR-968	uniq_723303	7.675957033	11.09912861	3.50891831	0.000509023	27	UUUGAACUGUGACAAUAGGAAGAUCC
ASY-piR-1415	uniq_548510	3.142957954	6.894817763	3.505699222	0.031177821	29	UUCGAUCUGUGACUUUAAGGUAAGCUACC
ASY-piR-452	uniq_379764	10.78299821	13.99246633	3.209468118	0.000163421	31	GACCACCUGGGAAUAUCAAGUGCUGCAGGAA
ASY-piR-935	uniq_1991592	7.272240971	10.40921412	3.136973154	0.000213761	31	UCCAGCUGAUUCAGCACGUGACCGGACCAGC
ASY-piR-897	uniq_1790231	6.723376529	9.724798044	3.001421515	0.003461984	28	UGGACUUGUUUGCAGAUCUGCAGGAUGC
ASY-piR-1671	uniq_898121	6.273018494	9.198240389	2.941887262	0.002472557	28	UCCAGCUGAUUCAGUACGUGACCGGACC
ASY-piR-696	uniq_338579	6.360481336	8.625708843	2.907085673	0.00216095	31	UCCAGCUGAUUCAGCACGUGACCGGACCAGU
ASY-piR-553	uniq_250665	6.115477217	8.779992878	2.869100352	0.00426826	28	UCCAGCUGAUUCAGCACGUGACCGGACC
ASY-piR-722	uniq_872600	5.888743249	8.669475648	2.818615883	0.001238356	29	UCCAGCUGAUUCAGCACGUGACCGGACCA
ASY-piR-347	uniq_619718	1.99770178	5.348728154	2.804834846	0.027935801	25	UUUGUCUUACUUCUGUCCAAUGAUA
ASY-piR-1053	uniq_894090	5.878561873	8.660590206	2.784106503	0.021705902	26	CGGAUUGUGGAGCCCUCCAGUGACGC
ASY-piR-1720	uniq_310483	6.456696651	8.251876859	2.761527573	0.007274778	26	UCAUCAUGUGACUCUUCAGAGUGAGC
ASY-piR-980	uniq_2027275	6.502500341	8.703903573	2.432722958	0.025478396	29	UGAUGUUGGGCUGCACGAUGCCCUGAGCA
ASY-piR-771	uniq_264839	8.471336514	10.7968508	2.383531869	2.32E-05	26	UGGACGUGCUGGUACCUGGGGACACC
ASY-piR-3	uniq_304406	6.206200388	8.549463819	2.343263432	0.00407124	27	UCUGCGUACAGGAAUCUCUGCAGGCAC
ASY-piR-468	uniq_789106	6.648657176	8.835524112	2.333023772	0.002487475	29	UUCAUUCACUAGACCUGCUCUGAACAAGC
ASY-piR-1574	uniq_401938	6.386581053	8.627837834	2.224115648	0.003820257	29	UGAACGCUGGUAGGGAACCUGGACCCUCC
ASY-piR-342	uniq_860961	10.69537331	12.85610126	2.183603586	0.000162304	27	GGGGAUGUAGCUCAGUGGUAGAGCGCA
ASY-piR-1482	uniq_27	6.539158811	8.54303182	2.178768169	0.015324725	27	UUCAUCGUGUGACUCUUCAGAGUGAGC
ASY-piR-356	uniq_513451	4.763765654	6.759333407	2.086908065	0.001217699	25	UAUUUGCAACUGUUGUCACAGGAAC

ASY-piR-8	uniq_552634	6.115477217	7.892289823	2.005624549	0.018833179	27	UCUGCGUACAGGUAUCUCUGCAGGCAC
ASY-piR-1091	uniq_446809	9.101756865	10.9944713	1.892714434	0.004365335	27	UCCGACUGUAAGGCAACAUCAUCCACC
ASY-piR-992	uniq_890500	8.9522559	10.54101593	1.888845335	0.001281159	26	UUACAUGAACUAGGACAGAACCCUUC
ASY-piR-911	uniq_379100	6.372139541	8.054378208	1.851057731	0.000594409	28	UGACGUCUCUGCACCAGCCAGAUGGCCA
ASY-piR-1723	uniq_396207	6.549463819	8.423466121	1.831208462	0.03003906	26	UGAUGACGAAGAGACUACUCGAAGCU
ASY-piR-1497	uniq_615839	6.996238081	8.820178962	1.823940881	0.038682257	26	UCUGCGUACAGGAAUCUCUGCAGGGC
ASY-piR-1775	uniq_109760	11.22811721	13.04732276	1.819205548	0.010993917	31	GCAUUUGUGGUUCAGUGGUAGAAUUCUCGCC
ASY-piR-780	uniq_379103	6.924812504	8.688250309	1.802716027	0.004287667	28	UGACGUCUCUGCACCAGCCAGAUGGCCC
ASY-piR-382	uniq_821654	5.398031074	7.183221824	1.78519075	0.000367175	27	UCCGGCUGGCAGAUCUCAUAAUCCACC
ASY-piR-1040	uniq_127062	7.577428828	9.32305476	1.762085115	0.014064128	26	UGUGGAUUUCCUUUCCAGUAGACACC
ASY-piR-63	uniq_94215	5.273018494	6.981091537	1.708073043	0.000294217	28	UGACCAGAGCUUAGCCAGAACUUAGCCU
ASY-piR-775	uniq_2036736	6.241585987	7.892289823	1.650703837	0.0033281	29	UGACGUCUCUGCACCAGCCAGAUGGACUG
ASY-piR-1189	uniq_622175	7.557144557	9.151862198	1.64232331	0.000210385	27	UGGACGUGCUGGUACCUGGGGACACCA
ASY-piR-297	uniq_811103	6.342815461	7.968666793	1.625851332	0.001828678	29	CGAUGAUGAUGAAGGUAACCUUACUGACG
ASY-piR-1315	uniq_298842	4.574908836	5.942514505	1.62058641	0.029278261	26	AGACUGUAGCUGGAUCCAUCUCCCAC
ASY-piR-565	uniq_538392	7.151439431	8.203348003	1.593786351	0.034513331	28	UGAUGUUGGGCUGCACGAUGCCCUGAGC
ASY-piR-1278	uniq_98191	8.786814144	10.49868303	1.551365106	0.005071774	25	UCUGUGGUGAUGGGAGAACAUCAUA
ASY-piR-848	uniq_731025	8.249508549	9.783271111	1.544771923	0.000425297	26	UGAAUCCCACUUCUGACACCACGUGU
ASY-piR-305	uniq_459398	4.837102265	6.156504486	1.532013621	0.018484887	28	UACCUAUGGAGACCAUCUGCAUCUGAGC
ASY-piR-134	uniq_314832	9.13442632	10.64835758	1.513931262	0.005543388	29	UCCGAUUCGGUCUGAAAAUACAAACCAGC
ASY-piR-1163	uniq_738150	11.73978061	13.10110025	1.509266272	0.000179455	28	GCAUUGGUGGUUCAGUGGUAGAAUUCUU
ASY-piR-1853	uniq_250294	7.460797161	8.587465008	1.498721861	0.002930814	26	UGACGUCUCUGCACCAGCCAGAUGGU
ASY-piR-604	uniq_387272	6.772589504	8.223197272	1.490747159	0.003993106	27	UGACGUCUCUGCACCAGCCAGAUGGCC
ASY-piR-138	uniq_732577	5.639039173	6.949340382	1.488100961	0.016415502	27	UGACCAGAGCUUAGCCAGAACUUAGCC
ASY-piR-375	uniq_7916	4.321928095	5.781359714	1.459431619	0.006560045	30	UCUUCGUACUGCACUGUGCCUACUAAAGGU
ASY-piR-807	uniq_387276	6.822305264	7.892289823	1.424684273	0.019407452	27	UGACGUCUCUGCACCAGCCAGAUGGCA
ASY-piR-1698	uniq_870172	7.941536738	9.098690097	1.399406848	0.043927263	29	UCCUGAUGCACACCAACCCUUGUCCUCCC
ASY-piR-1102	uniq_738146	11.84601267	12.76500977	1.390007262	0.022788885	28	GCAUUGGUGGUUCAGUGGUAGAAUUCUC
ASY-piR-1588	uniq_765050	5.014950341	6.26366044	1.389191322	0.03219886	26	UCUGGUAGAUCUUCUUACUCUCUGCA
ASY-piR-351	uniq_670142	4.044394119	5.431845787	1.387451668	0.046363884	29	UACAGAAUUCUCCAGACAGGCCUUGUGUC
ASY-piR-1706	uniq_66454	11.46258748	13.14749645	1.373463371	0.035277018	29	GCAUUGGUGGUUCAGUGGUAGAAUUCUCA
ASY-piR-30	uniq_825575	7.283860054	8.633600735	1.349740681	0.008754835	31	AGGCGAUGAUGAUGAAGGUAACCUUACUGAC
ASY-piR-54	uniq_774577	5.781359714	7.122396631	1.341036918	0.000523558	28	GAUGAUGAUGAAGGUAACCUUACUGACG
ASY-piR-429	uniq_42720	9.810571635	11.11829223	1.331960934	0.023683193	27	UGACUAUGUGAAGACCCUCUGCACAAC
ASY-piR-50	uniq_719498	13.25064385	14.57474134	1.324097487	0.015856868	29	UCUCGUCUGAUCUCAGAAGCUAAGCAACA
ASY-piR-1149	uniq_746849	7.228016971	8.525520809	1.297503839	0.010707308	27	UGAAACACGGUCUCUCUCUGAGCACAG
ASY-piR-1058	uniq_17123	7.181566408	8.279997414	1.290901199	0.024985751	28	UGGACGUGCUGGUACCUGGGGACACCAU
ASY-piR-336	uniq_758053	6.50779464	7.770388596	1.280809108	0.010775512	25	UCAGACAGCCUCUGUAGCAGACGGU
ASY-piR-51	uniq_42723	7.892795766	9.100224296	1.270644262	0.018748922	27	UGACUAUGUGAAGACCCUCUGCACAAU

ASY-piR-815	uniq_334052	11.43484538	12.87897689	1.26849882	0.011131668	29	GCAUUGGUGGUUCAGUGGUAGAAUUCUCG
ASY-piR-504	uniq_496422	11.18249781	12.78115477	1.19783424	0.031180419	29	UCCCAUAUGGUCUAGUGGUUAGGAUUCCU
ASY-piR-532	uniq_811101	6.227214805	7.407975835	1.180761031	0.010969513	29	CGAUGAUGAUGAAGGUAACCUUACUGACU
ASY-piR-1539	uniq_872471	7.848101264	8.960967268	1.166849077	0.022816103	29	UCAAUACUGUGGAGAGCCUGAUCUUUCUC
ASY-piR-1232	uniq_56024	9.8008999	10.95419631	1.15329641	0.003192227	28	UAGGAAUGGAAACAGACCAAGGACUUCU
ASY-piR-1810	uniq_891468	12.0832577	13.15429071	1.071033014	0.026132319	31	GCAUUGGUGGUUCAGUGGUAGAAUUCUCGUC
ASY-piR-203	uniq_627815	6.569855608	7.614709844	1.044854236	0.037967235	28	CGAUGAUGAUGAAGGUAACCUUACUGAC
ASY-piR-37	uniq_183930	8.533978572	9.752101838	1.031758216	0.023308314	30	UCCGAUUCGGUCUGAAAAUACAAACCAGCA
ASY-piR-1707	uniq_25448	7.189000868	8.223197272	1.029261297	0.02619858	26	UACUGUAGUCAUGAACCAGCUGACAC
ASY-piR-844	uniq_834130	7.112005026	8.122396631	1.010391606	0.002109874	29	UGACGUCUCUGCACCAGCCAGAUGGCCCG
ASY-piR-392	uniq_602422	4.709658248	5.714245518	1.004587269	0.041358187	26	UUCUUACCUGUUACUGCUGACCAGGA
ASY-piR-172	uniq_379120	6.440177062	7.64385619	1.004347653	0.01446724	29	CGAUGAUGAUGAAGGUAACCUUACUGGCA
